# Recover the activity of sintered supported catalysts by nitrogen-doped carbon atomization

**DOI:** 10.1038/s41467-019-14223-w

**Published:** 2020-01-17

**Authors:** Huang Zhou, Yafei Zhao, Jie Xu, Haoran Sun, Zhijun Li, Wei Liu, Tongwei Yuan, Wei Liu, Xiaoqian Wang, Weng-Chon Cheong, Zhiyuan Wang, Xin Wang, Chao Zhao, Yancai Yao, Wenyu Wang, Fangyao Zhou, Min Chen, Benjin Jin, Rongbo Sun, Jing Liu, Xun Hong, Tao Yao, Shiqiang Wei, Jun Luo, Yuen Wu

**Affiliations:** 1 Hefei National Laboratory for Physical Sciences at the Microscale, Collaborative Innovation Center of Chemistry for Energy Materials (iChEM), School of Chemistry and Materials Science, and National Synchrotron Radiation Laboratory, University of Science and Technology of China, Hefei, 230026 China; 2 Center for Electron Microscopy and Tianjin Key Lab of Advanced Functional Porous Materials, Institute for New Energy Materials & Low-Carbon Technologies, School of Materials Science and Engineering, Tianjin University of Technology, Tianjin, 300384 China; 3 Nano and Heterogeneous Materials Center, School of Materials Science and Engineering, Nanjing University of Science and Technology, Nanjing, 210094 China; 4 NEST Lab, Department of Chemistry, College of Science, Shanghai University, Shanghai, 200444 China; 5 National Synchrotron Radiation Laboratory, University of Science and Technology of China, Hefei, 230029 China; 6 Department of Chemistry, Tsinghua University, Beijing, 100084 China

**Keywords:** Chemical engineering, Nanoscale materials

## Abstract

The sintering of supported metal nanoparticles is a major route to the deactivation of industrial heterogeneous catalysts, which largely increase the cost and decrease the productivity. Here, we discover that supported palladium/gold/platinum nanoparticles distributed at the interface of oxide supports and nitrogen-doped carbon shells would undergo an unexpected nitrogen-doped carbon atomization process against the sintering at high temperatures, during which the nanoparticles can be transformed into more active atomic species. The in situ transmission electron microscopy images reveal the abundant nitrogen defects in carbon shells provide atomic diffusion sites for the mobile atomistic palladium species detached from the palladium nanoparticles. More important, the catalytic activity of sintered and deactivated palladium catalyst can be recovered by this unique N-doped carbon atomization process. Our findings open up a window to preparation of sintering-resistant single atoms catalysts and regeneration of deactivated industrial catalysts.

## Abstract (web summary)

Sintering supported metal nanoparticles is the common route to the deactivation of industrial heterogeneous catalysts. Here, the authors report a carbon atomization process that allows one not only to redisperse metal nanoparticles, but also to recover deactivated catalysts.

## Introduction

Supported metal catalysts can efficiently accomplish many important industrial applications in heterogeneous catalysis, including the production of chemicals^1^, pharmaceuticals^2^ and clean fuels^3^, and the purification of vehicle emissions^4–7^. However, most of the supported catalysts suffer from sintering, which significantly decrease their active surface areas, stability, and shut down the catalytic steps^6,8–11^. The sintering is usually accelerated above the Tammann temperature (half of the melting point in absolute units)^4,12^, involving the emission of mobile species from small particles and their capture by large particles (Ostwald ripening), or particles migration and coalescence^7,8,12^. This process is costly, particularly for those supported Earth-scarce metals such as Pt (refs. ^7,13–16^), Pd (refs. ^17,18^), Au (refs. ^5,10,19,20^), because the deactivated metal nanoparticles (NPs) should be replaced to satisfy the industrial-level efficiency. For example, replacing catalysts in a fuel cell stack would cost >50% expenditure due to the valuable price of Pt-based catalysts^3^. Therefore, there is primary interest in inspiring researchers in academia and industry to construct sintering-resistant catalysts and cut down the costs for catalyst regeneration and recovery.

Of note is that, increasing the metal-support interaction (MSI) has been widely utilized to remit the sintering propensity of supported metal catalysts^5,17,21–27^. One of the effective strategies to prevent particles sintering is confining metal NPs with porous supports, such as mesoporous alumina^4,28–30^, silica^20,21,26,31^, ceria^7^, or titanium^22,32^. Another typical strategy to enhance the stabilization of metal NPs on supports is to introduce strong ligands^33,34^. However, the steric hindrance of porous support and organic ligands on metal catalysts would block catalytic active sites and alter the activity and selectivity. Currently, two major challenges remain in the industry of supported metal catalysts: (i) preparation of catalysts that can resist sintering at high temperatures, and (ii) strategy to recover or regenerate the activity of sintered and deactivated catalysts.

In a recent interesting work^18^, Li et al. describe that the metal NPs can be transformed to thermally stable single atoms with assistence of nitrogen (N) defects at high temperatures. Inspired by this work, herein, we report a N-doped carbon atomization process to not only redisperse oxide supported metal NPs but also regenerate the sintered and deactivated supported metal catalysts. The N-doped carbon atomization process is opposite to the conventional sintering, during which the sintering of supported Pd/Au/Pt NPs on oxide supports is largely prohibited by covering the NPs with a N-doped carbon shell. This carbon shell containing abundant N defects allows the migration of atomic metal species detached from the large NPs at high temperatures, but impedes the large NPs. After the N-doped carbon atomization process, the as-obtained sintering-resistant Pd SAs exhibit superior activity for hydrogenation reactions with respect to Pd NPs.

## Results

**Fig. 1:**
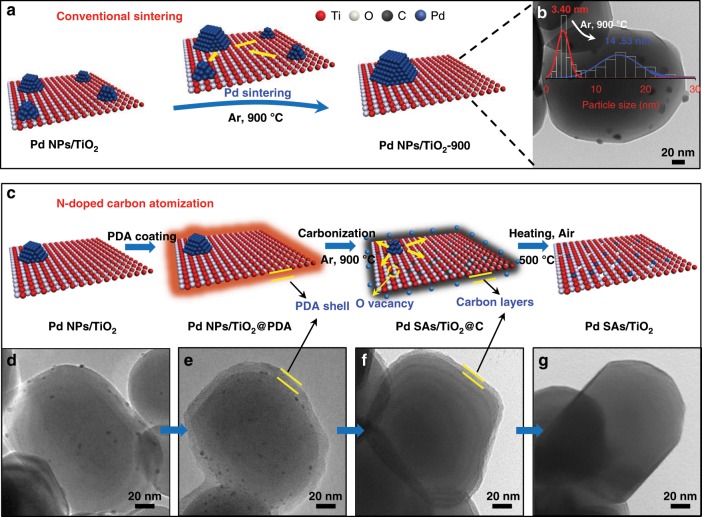
Schematic illustrations and TEM images for the preparation of Pd SAs/TiO_2_ and reference materials. **a** Schematic images of the conventional sintering of Pd NPs/TiO_2_, which results in larger Pd NPs/TiO_2_ (denoted as Pd NPs/TiO_2_-900). **b** TEM image of Pd NPs/TiO_2_-900, in which the inset contains the particle-size distributions of Pd NPs/TiO_2_ (red line) and Pd NPs/TiO_2_-900 (blue line). **c** Schematic images of the N-doped carbon atomization of Pd NPs/TiO_2_, which results in Pd SAs/TiO_2_. TEM images of (**d**) initial Pd NPs/TiO_2_, (**e**) Pd NPs/TiO_2_@PDA, (**f**) Pd SAs/TiO_2_@C, and (**g**) Pd SAs/TiO_2_. Scale bar, 20 nm.

The conventional sintering of Pd NPs supported on TiO_2_ is displayed in Fig. 1a. The Pd NPs homogeneously supported on the surface of TiO_2_ (denoted as Pd NPs/TiO_2_) aggregated to form large particles (from 3.40 to 14.53 nm) at 900 °C for 3 h (Fig. 1b; Supplementary Figs. 1, 2). By contrast, N-doped carbon atomization showed a totally different situation, in which small Pd NPs were transformed to atomically dispersed species (Fig. 1c–g). Specifically, a polydopamine (PDA) layer was deposited on the surface to form a core–shell structure (denoted as Pd NPs/TiO_2_@PDA, Fig. 1c; Supplementary Fig. 3), as evidenced by a typical stretching vibration for N/O–H at 3450 cm^−1^ and a bending vibration of N–H at 1615 cm^−1^ in Fourier-transformed infrared (FT-IR) spectroscopy^5^ (Supplementary Fig. 4). Although the color of Pd NPs/TiO_2_ changed from gray to dark brown (Supplementary Fig. 5), the particle size of Pd NPs retained as ~3.38 nm (Supplementary Fig. 3). Then, the PDA layer was transformed in situ to form N-doped carbon shells after carbonization at 900 °C under argon (Ar) atmosphere (Fig. 1f; Supplementary Fig. 6). The depressed signal for the organic species (N/O–H) in the FT-IR spectra and the appearance of characteristic D (1350 cm^−1^) and G (1580 cm^−1^) bands of carbon species in Raman spectra (Supplementary Figs. 4, 7) both traced this evolution. The coated N-doped carbon layer could serve as a “protector” to limit the migration of supported Pd NPs and their following aggregation. Meanwhile, TiO_2_ was reduced into defective TiO_2_ slowly, and the atomic-scale species emitted from supported Pd NPs diffused into the carbon layers (Fig. 1f; Supplementary Fig. 6), in which the abundant N defects are essential for the transformation of Pd NPs to SAs (Supplementary Figs. 8, 9). The specific chemical environments of N were investigated by X-ray photoelectron spectroscopy (XPS) and the near-edge X-ray absorption fine structures (NEXAFS) (Supplementary Fig. 10). The pyrolyzed N-doped carbon shells were determined to be 5.4 wt% by a thermogravimetric analysis (TGA) (Supplementary Fig. 11), and can be removed subsequently by a post-thermal treatment in air at 500 °C. This is accompanied with a dramatic color change from black to light yellow (Supplementary Fig. 5), also supported by the vanishment of carbon signals in FT-IR and Raman spectra (Supplementary Figs. 4, 7). To our surprise, though there is no conspicuous Pd NPs or clusters observed by the transmission electron-microscopy (TEM) measurements (Fig. 1g), the signals detected from the energy-dispersive X-ray spectroscopy (EDS) analysis and the inductively coupled plasma optical emission spectrometry (ICP-OES) measurements highlighted the existence of Pd species on TiO_2_ surface (Supplementary Fig. 12, Supplementary Table 1). Considering the high boiling point of Pd (mp 1555 °C, bp 2970 °C)^12^, it is reasonable to propose that the resultant Pd species are atomic scale and the transformation from NPs to SAs indeed took place.

**Fig. 2:**
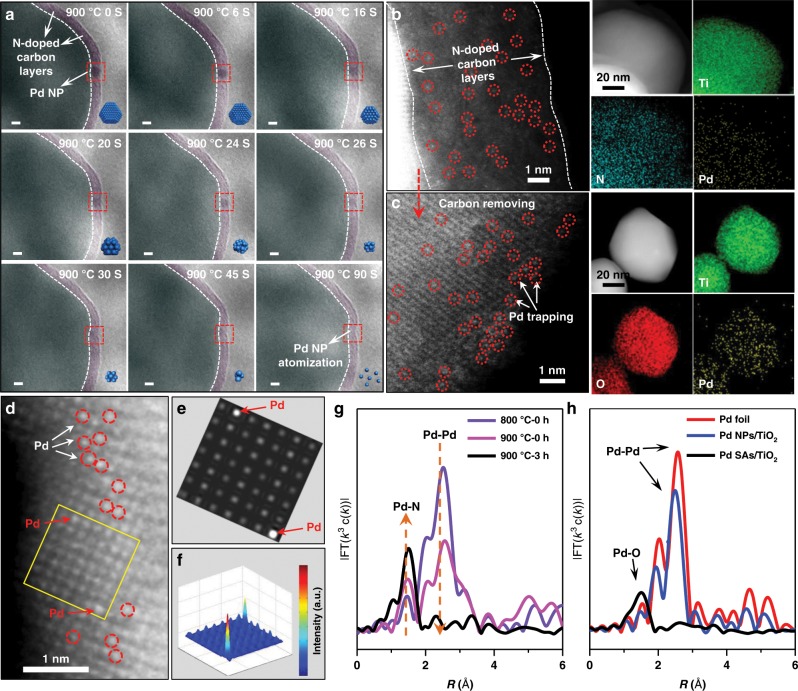
NP-to-SA transformation of Pd and corresponding structural characterizations. **a** Representative movie images of Pd NPs/TiO_2_@C acquired at 900 °C at different times with in situ TEM under Ar atmosphere. **b**, **c** Aberration-corrected HAADF-STEM images and EDS mapping images of Pd SAs/TiO_2_@C (**b**) and Pd SAs/TiO_2_ (**c**). The HAADF image in (**b**) was taken from a region similar to the boxed one in (**a**). AC HAADF-STEM image (**d**) of Pd SAs/TiO_2_ and the simulated image (**e**) obtained from the yellow rectangle in (**d**), and (**f**) the corresponding 3D surface intensity profile image. Some of the Pd SAs in the HAADF images of (**b**–**d**) are highlighted by red circles. **g** Fourier-transformed (FT) k^3^-weighted Pd K-edge EXAFS spectra for Pd NPs/TiO_2_@PDA during the annealing. **h** Fourier-transformed (FT) k^3^-weightedχ(k)-function of the EXAFS spectra for Pd K-edge of Pd SAs/TiO_2_ and Pd NPs/TiO_2._ Scale bar, 5 nm for (**a**), 1 nm for AC HAADF-STEM images in (**b**–**d**), 20 nm for mappings in (**b**, **c**).

To investigate the intrinsic mechanism of high temperature and N-doped carbon atomization, the evolution process from Pd NPs/TiO_2_@PDA to Pd SAs/TiO_2_@C was further traced by in situ TEM. Representative TEM images acquired at different temperatures (Supplementary Fig. 14) showed that no observable change occurred in size distribution and particle number of the supported Pd NPs when the temperature was lower than 800 °C, indicating the migration and aggregation were largely prohibited by the carbon layer. Interestingly, as the temperature was elevated over 800 °C, the N-doped carbon atomization would be initiated and accelerated with the temperature rise, resulting in a gradually decreasing particle number of supported Pd NPs (Supplementary Fig. 14). The Pd NPs owning smaller sizes exhibited a strong tendency to vanish due to the quick loss of atoms. As shown in the marginal area of Fig. 2a, the marked Pd NP with a diameter of 5.5 nm (at 0 s) was downsized to 4.8 nm at 16 s and to 2.6 nm at 30 s, and finally vanished at 90 s. This stems from the emission of surface Pd atoms and their continuous thermal diffusion within N-doped carbon layers and trapped by the N coordination. As shown in Fig. 2b and Supplementary Fig. 15a, the isolated high-density bright dots (highlighted by red circles) could be assigned to single-Pd atoms, demonstrating the migration of emitted Pd species within the N-doped carbon layers. EDS mappings further revealed that the Pd, N, and Ti were homogeneously dispersed over the whole architecture. The detailed N-doped carbon atomization process was recorded by the in situ TEM (Supplementary Movie 1). After removing the carbon shells, these isolated Pd atoms were subsequently captured by O defects on TiO_2_ support, as confirmed by the isolated brighter dots (Fig. 2c, d; Supplementary Fig. 15b). The simulation image (Fig. 2e) obtained from yellow rectangle in Fig. 2d and corresponding surface intensity profile (Fig. 2f) clearly showed the presence of Pd SAs, which is well consistent with the experimental STEM image. According to the Brunauer–Emmett–Teller (BET) and ICP-AES analysis (Supplementary Fig. 16, Supplementary Table 1), the surface coverage of Pd atoms in Pd SAs/TiO_2_@C and Pd SAs/TiO_2_ are estimated to be 0.58 and 1.14 atoms nm^−2^, respectively. Furthermore, the selected Ti−L and O−K electron energy-loss spectroscopy (EELS) spectra recorded at various positions in STEM image revealed the generation of O defects on TiO_2_ surface^35^ (Supplementary Fig. 17). Also, the evolution from Pd NPs to Pd SAs in the annealing process was traced by extended X-ray absorption fine structure (EXAFS, Fig. 2g), during which the increased peak of Pd–N and disappeared peak of Pd–Pd indicated the transformation from Pd NPs to Pd SAs. Functional theory (DFT) calculations were performed to simulate this N-doped carbon atomization (Supplementary Fig. 19). Furthermore, EXAFS was performed to further confirm that Pd species were atomically dispersed on the TiO_2_ support (Fig. 2g). The EXAFS spectra of Pd NPs (Pd NPs/TiO_2_) displayed a peak ~2.5 Å, which was attributed to Pd–Pd bond. For Pd SAs/TiO_2_, only one dominant peak around 1.5 Å was observed, which can be assigned to Pd–O bond. This verified the successful transformation from Pd NPs to SAs during the N-doped carbon atomization process.

**Fig. 3:**
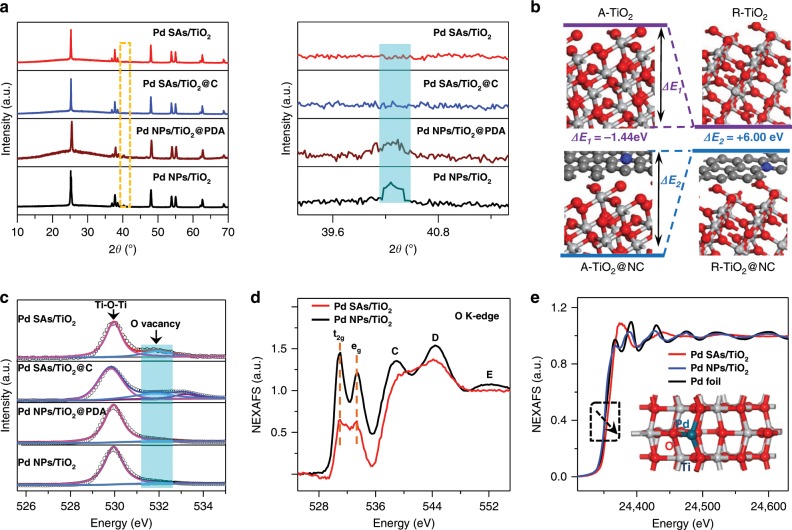
Structural characterizations of Pd SAs/TiO_2_ and reference materials. **a** XRD patterns. **b** Structure configurations and the energy difference of/between anatase TiO_2_ (A-TiO_2_) and rutile TiO_2_ (R-TiO_2_), anatase TiO_2_@N-doped carbon (A-TiO_2_@NC) and rutile TiO_2_@N-doped carbon (R-TiO_2_@NC). Silver white atoms represent Ti, red atoms represent O, gray atoms represent C and blue atoms represent N. **c** XPS spectra for samples during each synthetic step of N-doped C atomization. **d** O K-edge and **e** Pd K-edge NEXAFS spectra of Pd SAs/TiO_2_ and Pd NPs/TiO_2_. Inset is the proposed Pd–O_3_ architectures.

X-ray diffraction (XRD) patterns (Fig. 3a) displayed that a characteristic peak (2θ = ~40.3°) of Pd (111) disappeared after the carbonization (from Pd NPs/TiO_2_@PDA to Pd SAs/TiO_2_@C), indicating the evolution from well-crystallized Pd NPs to atomic species. Also, no observable signal of Pd crystals can be found for Pd SAs/TiO_2_, excluding the aggregates in the as-prepared Pd SAs samples. Interestingly, the coated carbon layers would change the normal phase evolution from anatase to rutile TiO_2_ along with the temperature (Supplementary Figs. 20–22). DFT calculations (Fig. 3b) reveal the total energy difference (*ΔE*) of the two types TiO_2_ is −1.44 eV, suggesting that the phase transition from anatase to rutile is an exothermic reaction. However, when the N-doped C (NC) layer was coated on anatase TiO_2_, the phase transition exhibits an endothermicity reaction with a larger *ΔE* of +6.00 eV, indicating that the phase transition of TiO_2_@NC needs more external energy. These results demonstrate that the coated carbon layers can delay the process of phase transition effectively. X-ray photoelectron spectroscopy (XPS) was used to track the change of characteristic O 1 s peaks (Fig. 3c), which is ascribed to the Ti–O–Ti and O-vacancy^19^. A higher peak area at 532 eV for Pd SAs/TiO_2_@C and Pd SAs/TiO_2_ with respect to the fresh supported samples demonstrated that more oxygen vacancies were constructed after annealing at high temperatures^36,37^. This coincides well with the smaller optical band gap of Pd SAs/TiO_2_ (3.17 eV) than Pd NPs/TiO_2_ (3.24 eV), which was observed by UV−vis diffuse reflectance spectra (Supplementary Fig. 23). Furthermore, more detailed structural information can be obtained from the near-edge X-ray absorption fine structure (NEXAFS). The O 1 s (k-edge) NEXAFS of Pd NPs/TiO_2_ showed almost identical spectra to that of pure TiO_2_ (Supplementary Fig. 24a). Specifically, there were two strong bands (t_2g_ and e_g_) in the low-energy range of the spectra (528–535 eV) for the oxygen 2p states that were hybridized with the empty split Ti 3d bands^38^. As for the C, D, and E peaks in the high-energy region, they were formed by the delocalized antibonding O 2p states and Ti 4sp band^39^. For Pd SAs/TiO_2_ (Fig. 3d), the intensity of the four peaks (t_2g_, e_g_, C, and D) were lower, and the E peak was absent. These suggest that the N-doped carbon atomization can create defects on the surface of supports. Supplementary Fig. 24b and c showed Ti 2p (L-edge) NEXAFS spectra of Pd NPs/TiO_2_, Pd SAs/TiO_2_, and pure TiO_2_ samples. There were two bands (L_3_ and L_2_) existing in each sample, presumably due to the 2p spin–orbit coupling^38^. In addition, L_3_ and L_2_ further split into *t*_2g_ and *e*_g_ as a consequence of the low symmetry of the TiO_6_^8−^ ligand field O_h_ (ref. ^38^). One small difference was visible for Pd SAs/TiO_2_: the L-edge absorption onset demonstrated a smaller red-shift than those of others, providing a preliminary evidence of a Ti oxidation state (< + 4) and the presence of oxygen vacancies near Ti atoms^19,40^. Electron paramagnetic resonance (EPR) spectrum of Pd SAs/TiO_2_ showed a prominent peak at 2.003 correspond to single electron-trapped oxygen vacancies, further suggesting the O defects in Pd SAs/TiO_2_ (Supplementary Fig. 25). The NEXAFS spectrometry of Pd SAs/TiO_2_ in Fig. 3e showed near-edge absorption energy more positive than those of Pd NPs/TiO_2_ and Pd foil, implying that the Pd single atoms carried positive charges. Also, by the XPS measurements, the binding energy of Pd 3d_5/2_ peak at 336.5 eV for Pd SAs/TiO_2_ was higher than that of Pd^0^ (335.3 eV) for Pd NPs/TiO_2_, suggesting the ionic Pd^δ+^ (0 < δ) nature of Pd in Pd SAs/TiO_2_ (Supplementary Fig. 26). Quantitative analysis of Pd SAs/TiO_2_ and Pd NPs/TiO_2_ were carried out through least-square EXAFS fitting. The fitting curves are displayed in Supplementary Figs. 27–29, and the corresponding structure parameters are listed in Supplementary Table 2. The first coordination number of the central atom Pd is about three and the average bond length of Pd–O is 1.97, whereas the coordination number of Pd NPs is about 9.7 and the bond length is 2.72, similar to those of Pd foil.

**Fig. 4:**
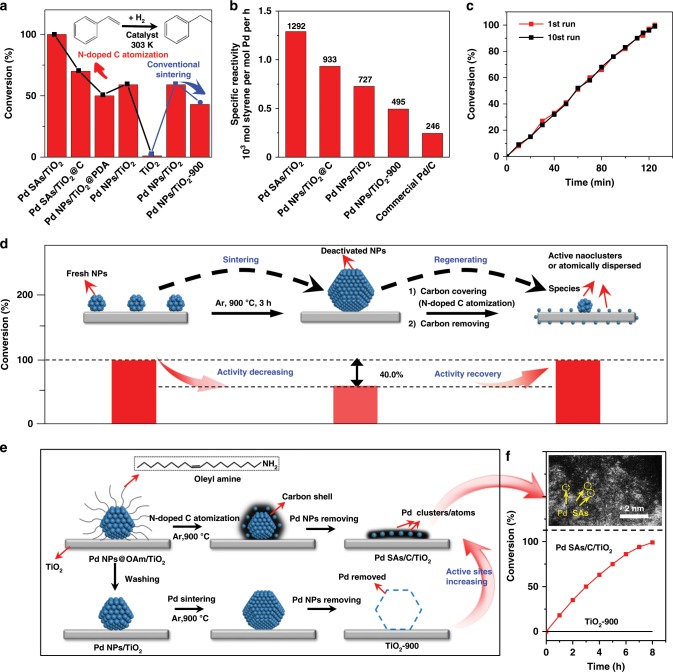
Catalytic performances and schematic illustrations of Pd SAs and Pd NPs. **a** Catalytic performance for fresh TiO_2_ and catalysts prepared by N-doped C atomization and conventional sintering in styrene hydrogenation. **b** Specific reactivity of Pd SAs/TiO_2_ and the reference catalysts. **c** Catalytic stability of Pd SAs/TiO_2_. **d** Schematic images (top) of the formation of deactivated NPs and active nanoclusters or atomic species. Catalytic performance (bottom) of Pd NPs/TiO_2_, Pd NPs/TiO_2_-900 and Pd NPs/TiO_2_-900 after treatment by N-doped C atomization process (denoted as Pd SAs/NPs/TiO_2_-900) in styrene hydrogenation for 3.5 h. **e** Schematic images of the formation of Pd SAs/C/TiO_2_ by N-doped C atomization process (by using Pd NPs@OAm/TiO_2_ as precursors) and TiO_2_-900 by conventional sintering (by using Pd NPs/TiO_2_ as precursors). **f** AC HAADF-STEM image of Pd SAs/C/TiO_2_ and its catalytic performance. Scale bar, 2 nm.

Hydrogenation reactions were subsequently conducted to further confirm the presence of atomically dispersed Pd species. As shown in Fig. 4a and Supplementary Fig. 30, the pure TiO_2_ showed <1% styrene conversion to phenylethane after 125 min of reaction. However, the Pd SAs/TiO_2_ delivered over 99% conversion during the same time span, indicating the highly active Pd species. With comparable metal loading, the Pd NPs/TiO_2_-900 delivered only 43% conversion, inferior to fresh Pd NPs/TiO_2_ (59%), revealing the activity loss caused by conventional thermal sintering. The specific reactivity (SR) for the prepared Pd species samples was further calculated to compare catalytic efficiency. As shown in Fig. 4b, the SR value of Pd SAs/TiO_2_ (1292 moles of styrene per mole of palladium per hour) was greater than those Pd NPs/TiO_2_ by a factor of 1.8, Pd NPs/TiO_2_-900 by a factor of 2.6, and Pd/C by a factor of 5.3, revealing the highest catalytic efficiency. Furthermore, another hydrogenation reaction of nitrobenzene also confirmed that the Pd SAs/TiO_2_ could reach a higher reaction rate than Pd NPs/TiO_2_-900. As shown in Supplementary Fig. 32, the Pd SAs/TiO_2_ showed a >99% nitrobenzene conversion in 150 min, whereas Pd NPs/TiO_2_-900 exhibited only 29% conversion under identical condition. Figure 4c and Supplementary Fig. 33 exhibited that the catalytic activity and reaction rate of Pd SAs/TiO_2_ were maintained even after ten cycles, suggesting the robust nature of the atomically dispersed Pd SAs/TiO_2_. As shown in the aberration-corrected HAADF-STEM and EXAFS images (Supplementary Fig. 34), the Pd species remained atomically dispersed after ten catalytic cycles. These results indicated that the atomically dispersed Pd species were strongly bounded to defective TiO_2_ through N-doped carbon atomization, and they exhibited excellent chemical and thermal stability. Furthermore, we used Fe^3+^ solution to etch the supported Pd species of Pd SAs/TiO_2_ and Pd NPs/TiO_2_ through an oxidative process^41^. The Pd SAs/TiO_2_ treated by Fe^3+^ solution exhibited only ~8% decrease in activity (Supplementary Fig. 35), indicating the robustness of atomically dispersed Pd species. By contrast, as evidenced by TEM image (Supplementary Fig. 36), the Fe^3+^ solution could etch nearly all of the supported Pd NPs for Pd NPs/TiO_2_, accompanied by a heavy activity loss (Supplementary Fig. 35).

To further study the activity and stability of the obtained Pd SAs, their catalytic performance was studied by the diphenylmethylsilane oxidation with water. As a result, the conversion of silane to silanol was >99% after 50 min catalysis reaction by atomically dispersed Pd species (Pd SAs/TiO_2_), while 18% conversion was delivered by supported Pd NPs with the same Pd loading (Supplementary Fig. 37). Importantly, no other products, i.e., siloxanes, were detected during the reaction catalyzed by Pd SAs/TiO_2_. Moreover, the Pd SAs also delivered an excellent stability and selectivity (>99%) throughout a five cycles recycling test. Similar results can be obtained by applying Pd SAs to catalyze hydrosilylation of 1-octene as a model substrate with triethoxysilane ((EtO)_3_SiH). As shown in Supplementary Fig. 38, the Pd SAs/TiO_2_ exhibited an excellent selectivity of >98% toward anti-Markovnikov addition product of triethoxy(octyl)silane with a conversion of >99% within 3 h. In comparison, the conversion catalyzed by supported Pd NPs reached only 32%. In addition, the activity and selectivity of the single-Pd atom catalyst was maintained (Supplementary Fig. 38). Overall, the above reactions all demonstrated that a type of more active and selective Pd SAs was generated by this N-doped carbon atomization process, compared with the initial supported Pd NPs catalyst.

Moreover, by our developed strategy, a severely sintered and deactivated Pd NPs catalyst could be reactivated by downsizing the supported Pd NPs and regenerating the more active atomically dispersed Pd species (Fig. 4d). TEM and HAADF-STEM images in Supplementary Figs. 2, 39, 40 showed that the sintered Pd NPs supported on TiO_2_ with an average size of 14.53 nm could be cut down to ~2 nm. This process also generated abundant atomically dispersed Pd sites (as verified by XRD, STEM and EXAFs images in Supplementary Figs. 40, 41). With comparable Pd loading, the conversion of sintered Pd NPs catalyst (60% in 3.5 h for hydrogenation reaction) could be recovered to >99% after the N-doped carbon atomization (Fig. 4d), accompanied by the specific reactivity recovery (from 525 h^−1^ to 756 h^−1^, Supplementary Fig. 42).

It is deduced that the N-doped carbon atomization is a more general phenomenon that can exist in various supported metal (Pd, Au, Pt) catalysts containing nitrogen ingredients. Similarly, small Au/Pt NPs could also be successful transformed into atomically dispersed Au/Pt sites due to the strong N coordination and trapped by O defects on TiO_2_ surface (Supplementary Figs. 43, 44). They have been studied as well, and will be presented in a subsequent paper. Also, Pd NPs with and without oleylamine (OAm) coating were prepared and supported on TiO_2_ (Fig. 4e; Supplementary Figs. 45–47). Followed by pyrolysis at 900 °C, the OAm shells would be transformed in situ to N-doped carbon, which would provide the atomic diffusion sites for the migration of atomic Pd species at high temperatures (as demonstrated by inset AC HAADF-STEM images in Fig. 4f and Supplementary Fig. 48). Etched by the Fe^3+^ solution, the Pd NPs without the protection of OAm were successfully removed, leaving an inert TiO_2_ support. Interestingly, the etched OAm-capped Pd catalyst was still active for the hydrogenation reactions, demonstrating the generation of the active and stable atomically dispersed Pd residues (Fig. 4f).

## Discussion

In summary, we discovered an unusual transformation process from Pd NPs to more active atomically dispersed Pd species through N-doped carbon atomization. Our findings unravel the connection between nanoscale and atomic-scale species at high temperatures. This may also open an avenue for preparing sinter-resistant single-metal atoms for various applications or recovering the sintered and deactivated industrial supported metal catalysts.

## Methods

### Chemicals

All chemicals were used as received without further purification. Sodium hydrate (NaOH), ethanol, toluene, acetone, hexane, ferric chloride (FeCl_3_·6H_2_O), tris(hydroxymethyl) aminomethane, styrene, nitrobenzene, resorcino, formaldehyde, and sodium carbonate were purchased from purchased from Sinopharm Chemical Reggent Co., Ltd. Dopamine-HCl was purchased from Beijing HWRK Chem Co., Ltd. TiO_2_ was purchased from Shanghai Macklin Biochemical Co., Ltd. Sodium tetrachloropalladate(II) > = 99.99% trace metals basis (Na_2_PdCl_4_), Pd(acac)_2_ (34.7%), chloroauric acid (HAuCl_4_·4H_2_O), Potassium hexachloroplatinate, 40% Pt (K_2_PtCl_6_) oleylamine(OAm), acetic acid, and borane tributylamine complex (BTB) were purchased from Sigma-Aldrich. Triethoxysilane, diphenylmethylsilane, and 1-Octene were purchased from TCI. Deionized water was used throughout this study.

### Preparation of Pd NPs/TiO_2_

First, 20 mg of Na_2_PdCl_4_ was added in 60 mL of H_2_O in a vial and sonicated for 30 min. After heating at 70 °C, 1 g of TiO_2_ was added, and the slurry was magnetically stirred for 12 h. The suspension was separated by centrifugation and dried at 60 °C under vacuum and then calcined at 300 °C in Ar for 2 h.

### Preparation of Pd NPs/TiO_2_@PDA

The pre-synthesized Pd NPs/TiO_2_ (500.0 mg) were dispersed by ultrasound in 200.0 mL of freshly prepared Tris buffer solution (10 mM, pH 8.5). Then 20.0 mL of dopamine-HCl (300.0 mg) solution was added to the buffer solution^42^. The mixed solution was allowed to stir for 12 h at room temperature. The resulting products were washed with deionized water and ethanol three times, and collected by centrifugation. After dried at 70 °C in a vacuum, the desired Pd NPs/TiO_2_@PDA was obtained.

### Preparation of Pd SAs/TiO_2_@C

In a typical procedure, the Pd NPs/TiO_2_@PDA power was transferred into a ceramic boat and placed in a tube furnace. The sample was annealed at 900 °C for 3 h under Ar atmosphere with a heating rate of 5 °C min^−1^. The obtained materials were denoted as Pd SAs/TiO_2_@C.

### Preparation of Pd SAs/TiO_2_

The Pd SAs/TiO_2_ were synthesized by directly heating the Pd SAs/TiO_2_@C in air at 500 °C for 2 h with a ramp rate of 5 °C min^−1^ before cooled down to room temperature naturally.

### Preparation of Au/Pt SAs/TiO_2_

The synthetic procedure of Au/Pt SAs/TiO_2_ was similar to Pd SAs/TiO_2_, except for using 10 mg of HAuCl_4_·4H_2_O or 20 mg of K_2_PtCl_6_ to replace 20 mg of Na_2_PdCl_4._ Also, Au/Pt NPs/TiO_2_@PDA was annealed at 1000 °C for 6 h under Ar atmosphere to obtained Au/Pt SAs/TiO_2_TiO_2_@C.

### Preparation of Pd NPs/TiO_2_-900

The synthetic procedure of Pd NPs/TiO_2_-900 was similar to Pd SAs/TiO_2_@C, except for using Pd NPs/TiO_2_ as precursors instead of Pd NPs/TiO_2_@PDA.

### Preparation of Pd SAs/NPs/TiO_2_-900

The synthetic procedure of Pd SAs/NPs/TiO_2_-900 was similar to Pd SAs/TiO_2_, except for using Pd NPs/TiO_2_-900 as precursors instead of Pd NPs/TiO_2_.

### Preparation of Fe^3+^ treated-Pd NPs/TiO_2_-900 and Fe^3+^ treated-Pd SAs/TiO_2_

Fe^3+^treated-Pd NPs/TiO_2_-900 and Fe^3+^treated-Pd SAs/TiO_2_ catalysts were obtained by dispersing Pd NPs/TiO_2_-900 and Pd SAs/TiO_2_ in an aqueous solution containing FeCl_3_ and HCl (pH = 0.55)^41^. In a standard procedure, 300 mg of KBr, 30 mg of FeCl_3_·6H_2_O, 0.18 mL of HCl, and 7 mL of DI water were mixed in a glass vial. The mixture was heated to 100 °C in an oil bath under magnetic stirring. Subsequently, 20 mg of Pd NPs/TiO_2_-900 or Pd SAs/TiO_2_ in 2 mL of  H_2_O was added. After stirring for 3 h, the product was collected by centrifugation, washed twice with ethanol and three times with water. After dried at 70 °C in a vacuum, the product was obtained.

### Preparation of Pd NPs@OAm

The synthetic route of Pd SAs/CN/TiO_2_ was followed the method reported by Sun’s group^43^. In typically, under a nitrogen flow, 75 mg of Pd(acac)_2_ was mixed in 15 mL of oleylamine (OAm). The formed solution was heated to 60 °C in 10 min, resulting in anear colorless solution. In all, 300 mg of borane tributylamine complex (BTB) was solvated in OAm (~3–4 mL), and quickly injected into the Pd-OAm solution. The color of solution was changed into brown-black immediately. The as-obtained solution was heated to 90 °C (3 °C/min), and kept at 90 °C for 60 min. The solution was cooled down to room temperature. The product was separated by centrifugation (8000 rpm for 8 min). Eventually, the product was dispersed in hexane with a concentration of 1.5 mg/mL.

### Preparation of Pd NPs@OAm/TiO_2_

In total, 1 g of commercial TiO_2_ was dispersed into above-mentioned Pd NPs@OAm solution (20 mL). Then, the resulting colloidal mixture was sonicated for 6 h (to ensure complete adherence of Pd NPs@OAm onto TiO_2_ support). After evaporation of hexane, the Pd NPs@OAm/TiO_2_ was obtained.

### Preparation of Pd NPs/C/TiO_2_

The synthetic procedure of Pd NPs/CN/TiO_2_ was similar to Pd SAs/TiO_2_@C, except for using Pd NPs@OAm/TiO_2_ as precursors instead of Pd NPs/TiO_2_ @PDA.

### Preparation of Pd SAs/C/TiO_2_

Pd SAs/C/TiO_2_ was prepared by removing the Pd NPs of Pd NPs/C/TiO_2_ using an aqueous solution containing FeCl_3_ and HCl (pH = 0.55). In a standard procedure, 300 mg of KBr, 30 mg of FeCl_3_·6H_2_O, 0.18 mL of HCl, and 7 mL of DI water were mixed in a glass vial. The mixture was heated to 100 °C in an oil bath under magnetic stirring. Subsequently, 40 mg Pd NPs/C/TiO_2_ was added. After stirring for 3 h, the product was collected by centrifugation, washed twice with ethanol and three times with water. After dried at 70 °C in a vacuum, the Pd SAs/C/TiO_2_ was obtained.

### Preparation of Pd NPs/TiO_2_ without OAm

In all, 10 mL of acetic acid was added to 40 mg of Pd NPs@OAm/TiO_2_ in 50 mL of hexane dispersion and heated at 70 °C for 10 h. Then the mixture was cooled down to room temperature. In total, 30 mL of ethanol was added, and the mixture was centrifuged at 8000 rpm for 8 mins. This procedure was repeated twice. After dried at 70 °C in a vacuum, the Pd NPs/TiO_2_ was obtained.

### Preparation of TiO_2_-900

The synthetic procedure of TiO_2_-900 was similar to Pd SAs/C/TiO_2_, except for using Pd NPs/TiO_2_ without OAm as precursor instead of Pd NPs@OAm/TiO_2_.

### Preparation of Pd NPs/TiO_2_-H_2_

In total, 20 mg of Na_2_PdCl_4_ was added in 60 mL of H_2_O in a vial, and sonicated for 30 min. After heating at 70 °C, 1 g of TiO_2_ was added, and the slurry was magnetically stirred for 12 h. The suspension was separated by centrifugation and dried at 60 °C under vacuum and then calcinated in air at 400 °C for 2 h and calcinated in 10% H_2_/Ar at 400 °C for 2 h.

### Preparation of Air-Pd NPs/TiO_2_ and Air-Pd NPs/TiO_2_-900

The Pd NPs/TiO_2_ and Pd NPs/TiO_2_-900 were calcinated in air at 500 °C for 3 h to obatin Air-Pd NPs/TiO_2_ and Air-Pd NPs/TiO_2_-900, respectively.

### Preparation of Pd NPs/TiO_2_@RF

Resorcino (10 mg), formaldehyde solution (10 μL, 37 wt%), and Pd NP/TiO_2_ (10 mg) were dispersed in 5 mL of ethanol/water solution (v/v 1:1) with sodium carbonate aqueous solution (10 μL, 5 mg/mL) as a catalyst^44,45^. After that, the solution was heated to 100 °C and kept at that temperature for 24 h. Finally, the obtained Pd NPs/TiO_2_@RF were collected and purified with distilled water by centrifugation.

### Preparation of Pd NPs/TiO_2_@C

In a typical procedure, the Pd NPs/TiO_2_@RF power was transferred into a ceramic boat and placed in a tube furnace. The sample was annealed at 900 °C for 3 h under Ar atmosphere with a heating rate of 5 °C min^−1^. The obtained materials were denoted as Pd NPs/TiO_2_@C.

### Characterization

XRD measurements were recorded on a Rigaku Miniflex-600 operated at 40 kV voltage and 15 mA current using a Cu Kα radiation (λ = 0.15406 nm) at a step width of 2° min^−1^. TEM images were recorded on a Hitachi-7650 worked at 100 kV. The high-resolution TEM and the corresponding EDS were recorded on JEOL JEM-2100F field-emission electron microscope at 200 kV. HAADF-STEM images and the corresponding EDS and EELS were recorded on a FEI Titan Cubed Themis G2 300 with a probe corrector at 200 kV. The BET (Brunauer–Emmett–Teller) test was obtained from micromeritics ASAP 2020 HD88 PLUS. All the samples were degassed at 573 K for 3 h. The pore size distribution was calculated from the HK and BJH method for micropores and mesopores, respectively. XPS experiments were performed at the Catalysis and Surface Science End station at the BL11U beamline of National Synchrotron Radiation Laboratory (NSRL) in Hefei, China. Thermogravimetric analyses (TGA) were carried out on a TA SDT Q600 thermal analyzer heating from room temperature to 900 °C at the rate of 5 °C min^−1^. Elemental analysis of Pd in the solid samples was detected by inductively coupled plasma atomic emission spectrometry (Optima 7300 DV). Fourier-transformed infrared resonance (FT-IR) spectra were obtained in transmission modeon a Nicolet 8700 FT-IR. Raman shifts were carried out by using a LabRAM Aramis Raman spectrometer instrument with an excitation wavelength of 514 nm using the Ar ion laser.

The in situ TEM study was performed on a FEI Talos F200X microscope operated at 200 kV and an in situ gas holder (DENSsolutions Climate S3 Plus) filled with 1 bar Ar (99.9995%).

Soft X-ray absorption spectra (Soft-XAS, C K-edge, and N-K-edge) were carried out at BL12B X-ray Magnetic Circular Dichroism (XMCD) station and BL10B photoemission end station of National Synchrotron Radiation Laboratory (NSRL, Hefei in China) in TEY mode. The samples were coated on double-sided carbon tape for characterization. Hard XAFS measurement and data analysis: XAFS spectra at the Pd K-edge were recorded at the XAS station (BL14W1) of the Shanghai Synchrotron Radiation Facility (SSRF), China. The Pd K-edge XANES data were recorded in a fluorescence mode. Pd foil and PdCl_2_ were used as references. The storage ring was working at the energy of 3.5 GeV. The hard X-ray was monochromatized with Si (111) double-crystals. The acquired EXAFS data were extracted and processed according to the standard procedures using the ATHENA module implemented in the IFEFFIT software packages.

### Catalytic evaluation

The hydrogenation reactions were operated using 25 mL Schlenk glass vessel tubes under 0.1 MPa H_2_ atmosphere. Specifically, different catalysts (Pd NPs/TiO_2_, Pd NPs/TiO_2_@PDA, Pd SAs/TiO_2_@C, Pd SAs/TiO_2_, Pd NPs/TiO_2_-900, Pd NPs/SAs/TiO_2_-900) and 160 μmol of nitrobenzene/styrene with a mole ratio of 1:2300 (Pd: nitrobenzene/styrene) were dispersed in 5 mL of ethanol. For TiO_2_-900 and Pd SAs/C/TiO_2_, 20 mg of catalysts and 80 μmol of styrene were dispersed in 5 mL of ethanol. Then H_2_ was used to completely remove air from the reactor and stirring at 303 K with different time in an oil bath. For silane oxidation reactions, 0.5 mmol of silane, Pd species catalysts (Pd SAs/TiO_2_, 10 mg; Pd NPs/TiO_2_, 10.6 mg), 0.1 mL of H_2_O and 2 mL of acetone were added to a 10 -mL round bottom flask. After stirring the result mixture under room temperature for a proper time, the catalyst was removed through filtration. For alkene hydrosilylation reactions, 1-Octene (1 mmol), triethoxysilane (1.1 mmol), Pd species catalysts (Pd SAs/TiO_2_, 15 mg; Pd NPs/TiO_2_, 16 mg), and toluene (3 mL) were sequentially added in a 20 -mL standard Schlenk tube, then the mixture was heated at 90 °C and stirred for a proper time. Conversion for all reactions is determined by gas chromatography (GC) analysis with octadecane as an internal standard. Selectivities are determined by GC-MS analysis.

## Supplementary information

Supplementary Information

Description of Additional Supplementary Files

Supplementary Movie 1

## Data Availability

The main data supporting the findings of this study are available within the article and its Supplementary Information. Extra data are available from the corresponding authors upon request.
