# Supplementary material for: Recover the activity of sintered supported catalysts by nitrogen-doped carbon atomization
Source: Nat Commun. 2020 Jan 17;11:335. doi: 10.1038/s41467-019-14223-w (PMC6969067; doi:10.1038/s41467-019-14223-w)
Supplement: Supplementary file 1 — Supplementary Information [file 41467_2019_14223_MOESM1_ESM.pdf]

Supplementary Information

Recover the activity of sintered supported catalysts by N-doped carbon atomization

Supplementary Figures and Tables (2-52)

Supplementary Notes (53-57)

Supplementary Methods (58-60)

Supplementary References (61-62)

Supplementary Figures and Tables

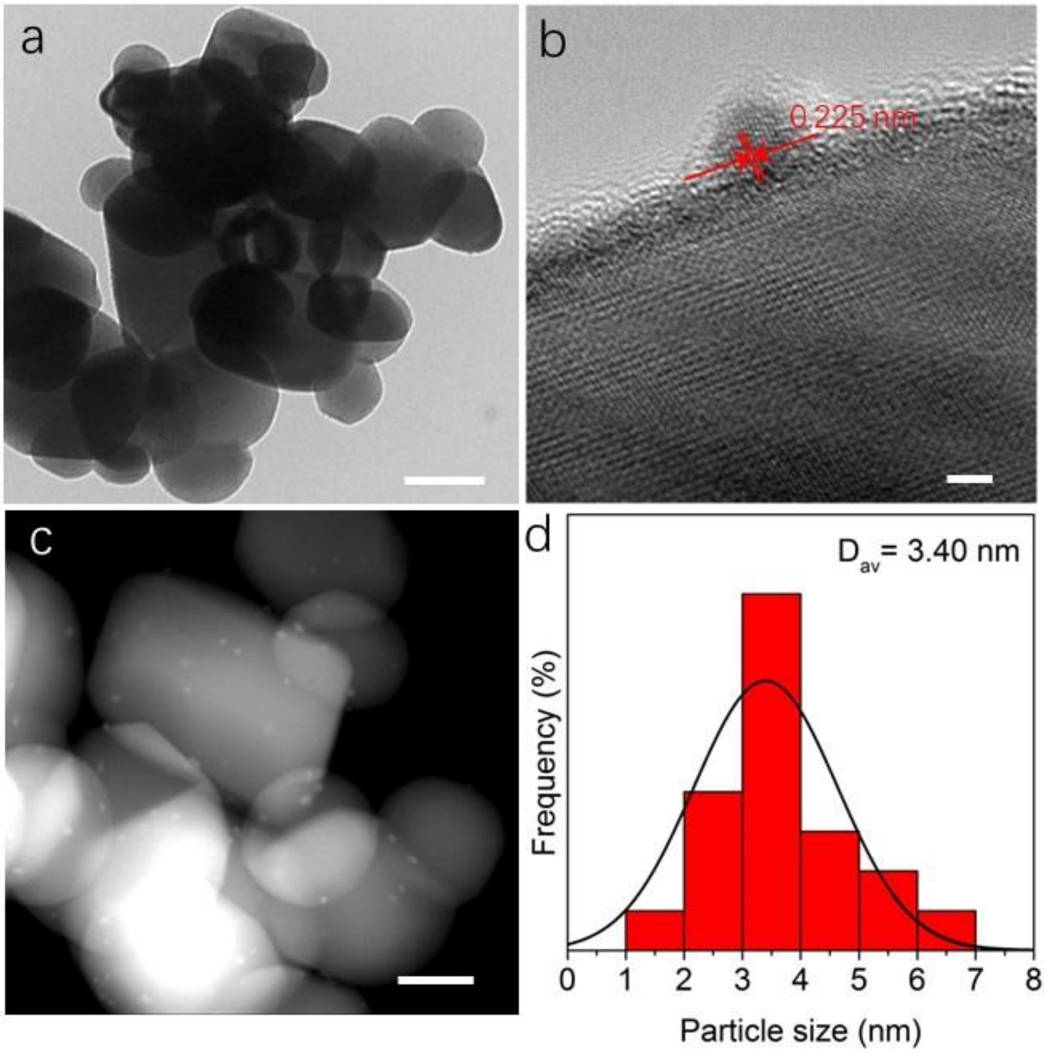

Supplementary Figure 1. Morphology characterization of Pd NPs/TiO_2. (a) TEM image. Scale Bar, 100 nm. (b) HRTEM image. Scale Bar, 2 nm. (c) HAADF-STEM image. Scale Bar, 50 nm. (d) Particle-size distribution.

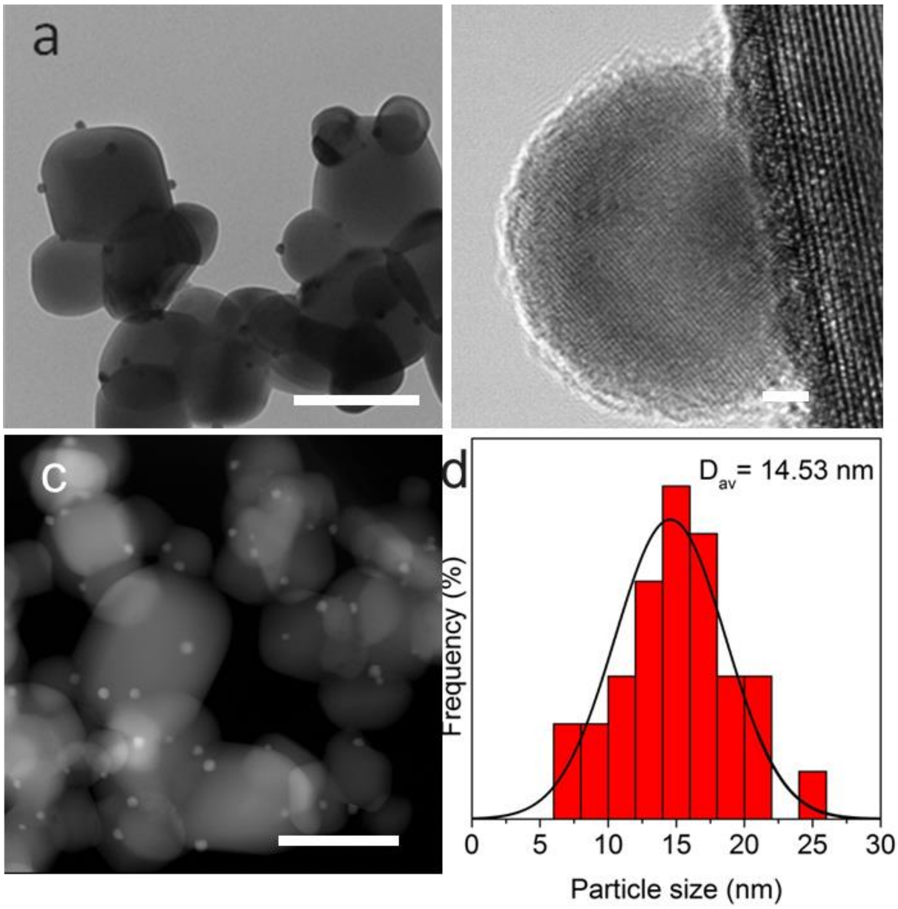

Supplementary Figure 2. Morphology characterization of Pd NPs/TiO_2-900. (a) TEM image. Scale bar, 200 nm. (b) HRTEM image. Scale bar, 2 nm. (c) HAADF-STEM image. Scale bar, 200 nm. (d) particle-size distribution.

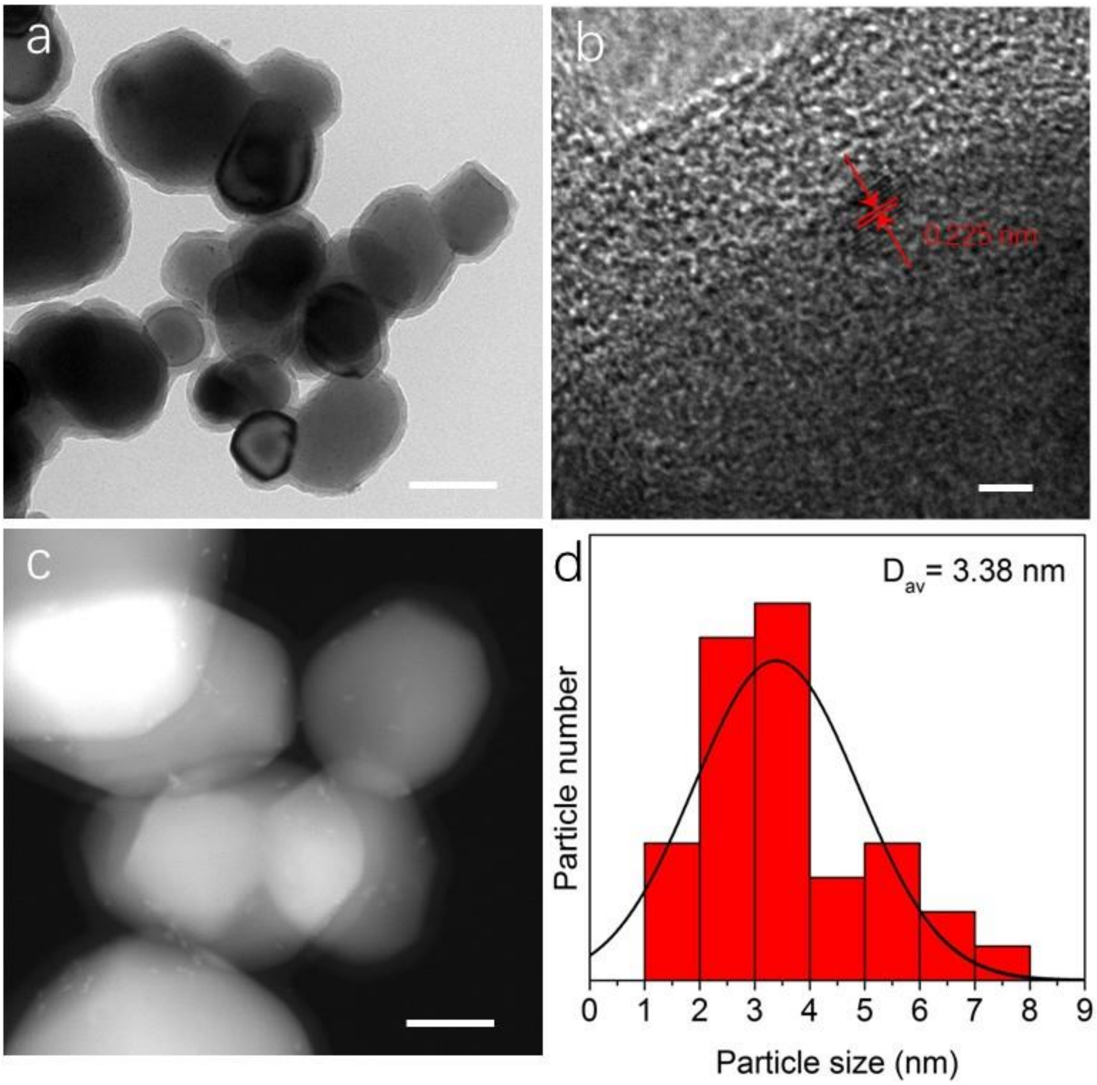

Supplementary Figure 3. Morphology characterization of Pd NPs/TiO_2@PDA. (a) TEM image. Scale Bar, 100 nm. (b) HRTEM image. Scale Bar, 2 nm. (c) HAADF-STEM image. Scale Bar, 50 nm. (d) particle-size distribution.

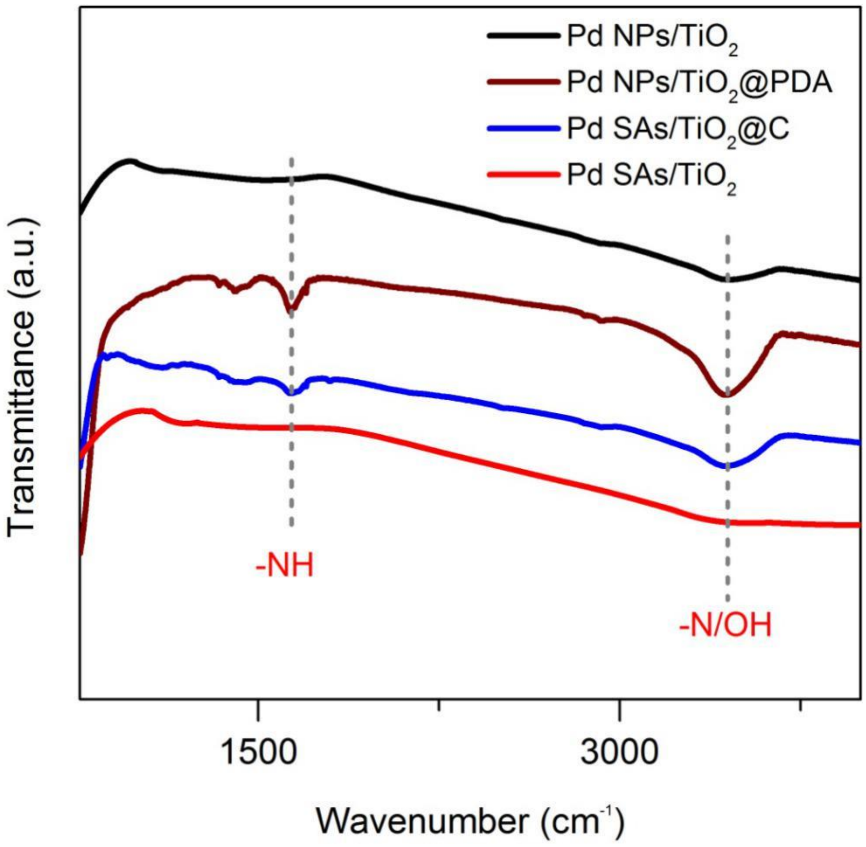

Supplementary Figure 4. FT-IR spectra of Pd NPs/TiO_2, Pd NPs/TiO_2@PDA, Pd SAs/TiO_2@C, and Pd SAs/TiO_2.

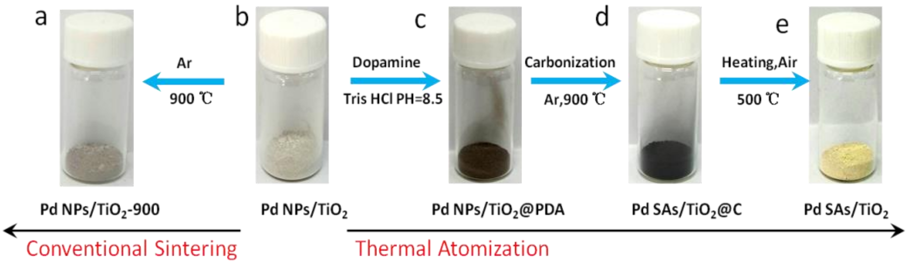

Supplementary Figure 5. Photographs of obtained samples by conventional sintering and thermal atomization. (a) Pd NPs/TiO_2-900, (b) Pd NPs/TiO_2, (c) Pd NPs/TiO_2@PDA, (d) Pd SAs/TiO_2@C, and (e) Pd SAs/TiO_2.

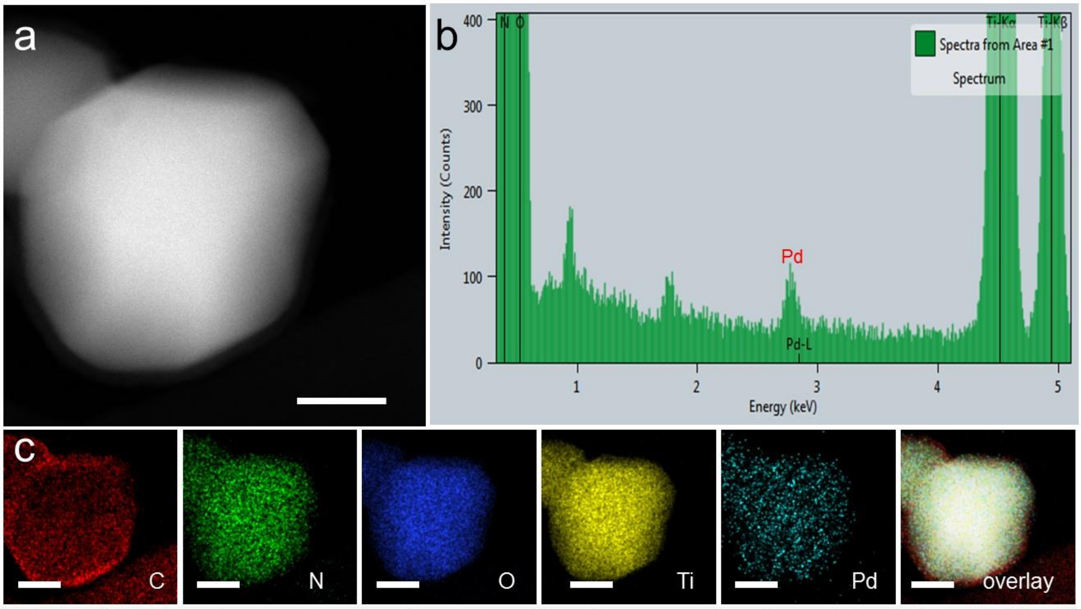

Element	Family	Atomic Fraction (%)	Atomic Error (%)	Mass Fraction (%)	Mass Error (%)	Fit Error (%)
C	K	10.65	1.95	8.85	0.86	0.49
N	K	6.50	1.78	3.39	1.07	22.50
O	K	46.46	12.48	27.15	6..06	2.00
Ti	K	36.05	8.01	60.31	9.92	0.11
Pd	L	0.33	0.08	0.30	0.07	14.63

Supplementary Figure 6. Morphology characterization of Pd SAs/TiO_2@C. (a) HAADF-STEM image, (b) EDS spectra, and (c) EDS mappings and data, showing that Pd nanoparticles were absent, and C, N, O, Ti, and Pd were homogeneously dispersed over the whole architecture. Scale bar, 50 nm.

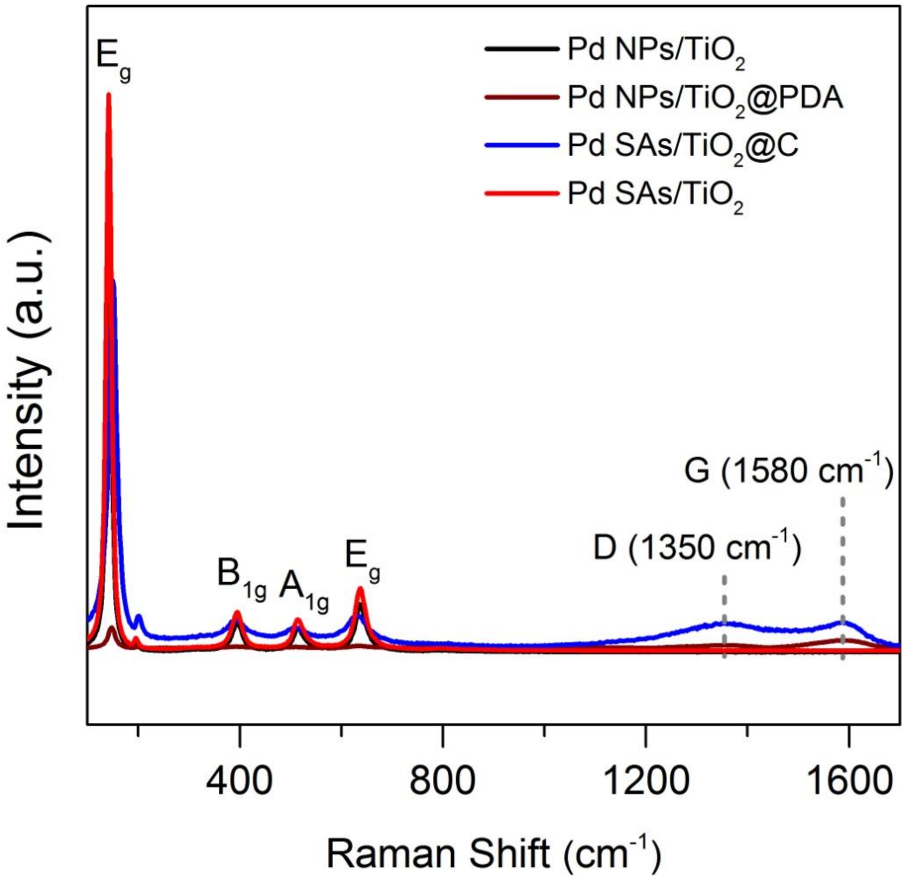

Supplementary Figure 7. Raman spectra of Pd NPs/TiO_2, Pd NPs/TiO_2@PDA, Pd SAs/TiO_2@C, and Pd SAs/TiO_2.

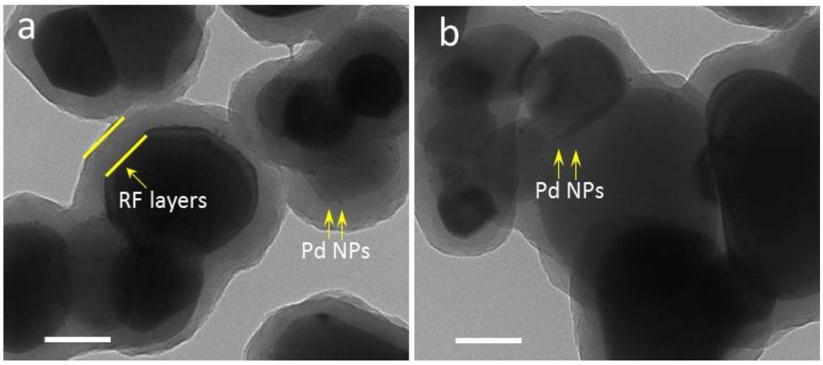

Supplementary Figure 8. TEM images of Pd NPs/TiO_2@RF. Scale bar, 100 nm.

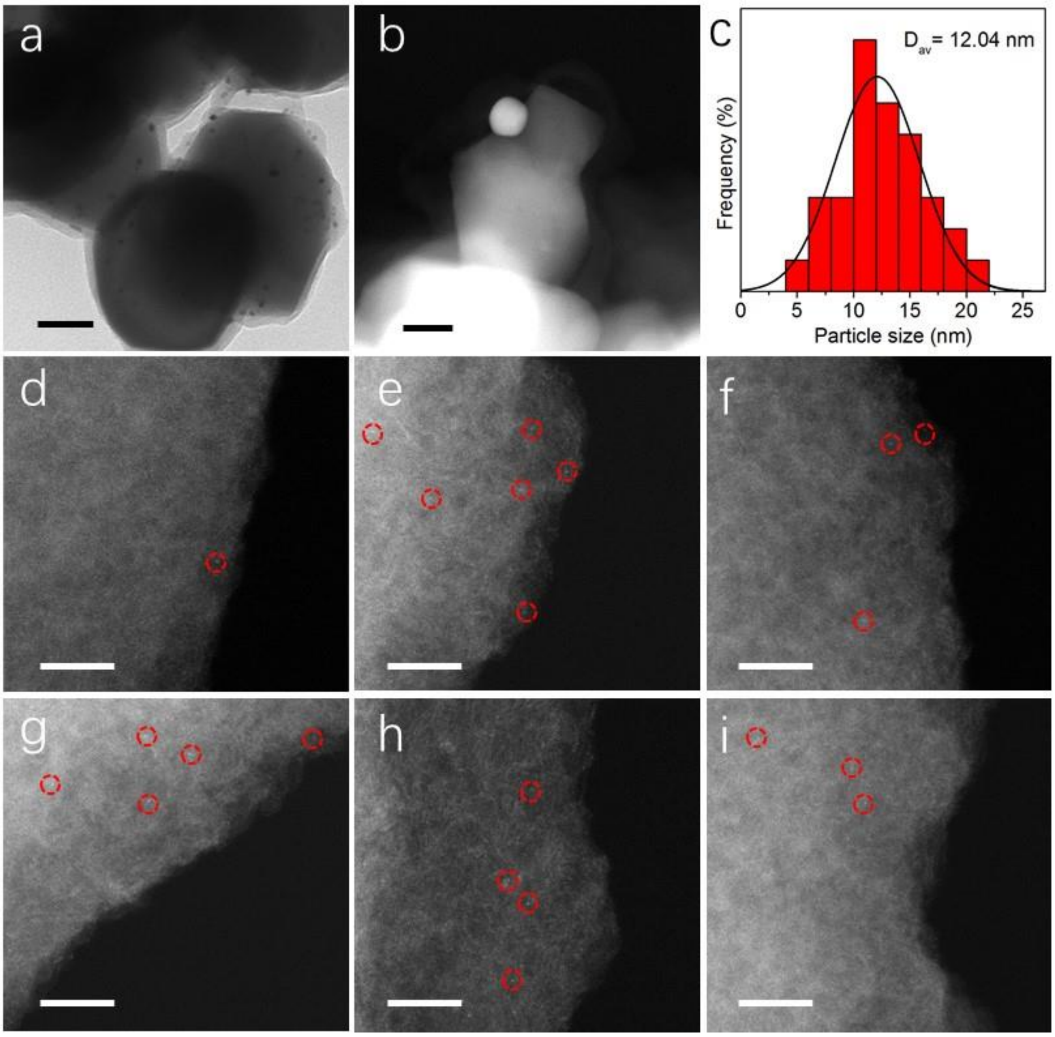

Supplementary Figure 9. Morphology characterization of Pd NPs/TiO_2@C. (a) TEM image. Scale bar, 100 nm. (b) HAADF image. Scale bar, 20 nm. (d-i) AC HAADF-STEM images. Scale bar, 5 nm. (c) Particle-size distribution of sintered Pd nanoparticles on TiO_2.

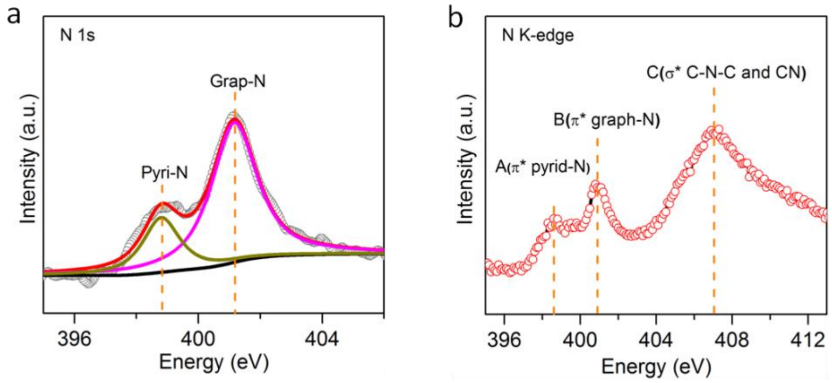

Supplementary Figure 10. Structure characterization of N in Pd SAs/TiO_2@C.
(a) XPS and (b) NEXAFS spectra.

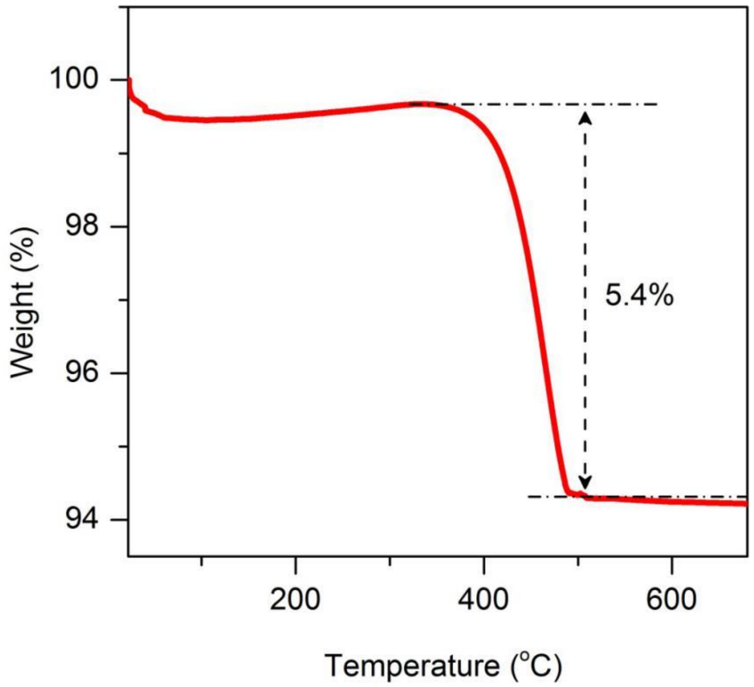

Supplementary Figure 11. Thermogravimetric analysis curve of Pd SAs/TiO_2@C.

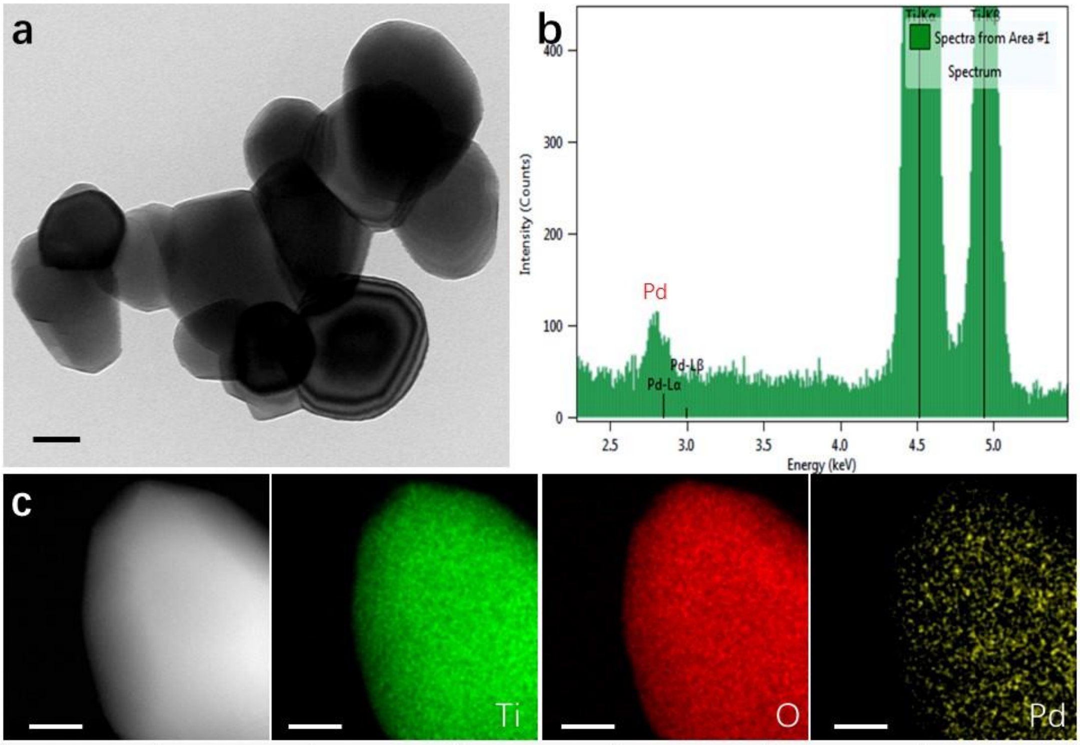

Element	Family	Atomic Fraction (%)	Atomic Error (%)	Mass Fraction (%)	Mass Error (%)	Fit Error (%)
O	K	62.81	8.16	35.96	3.07	1.37
Ti	K	36.84	6.85	63.30	9.96	0.72
Pd	L	0.35	0.04	0.74	0.15	13.63

Supplementary Figure 12. Morphology characterization of Pd SAs/TiO_2. (a) TEM image, (b) EDS spectra, and (c) EDS mappings and data, showing that Pd nanoparticles were absent, O, Ti, and Pd were homogeneously dispersed over the whole architecture. Scale bar, 50 nm.

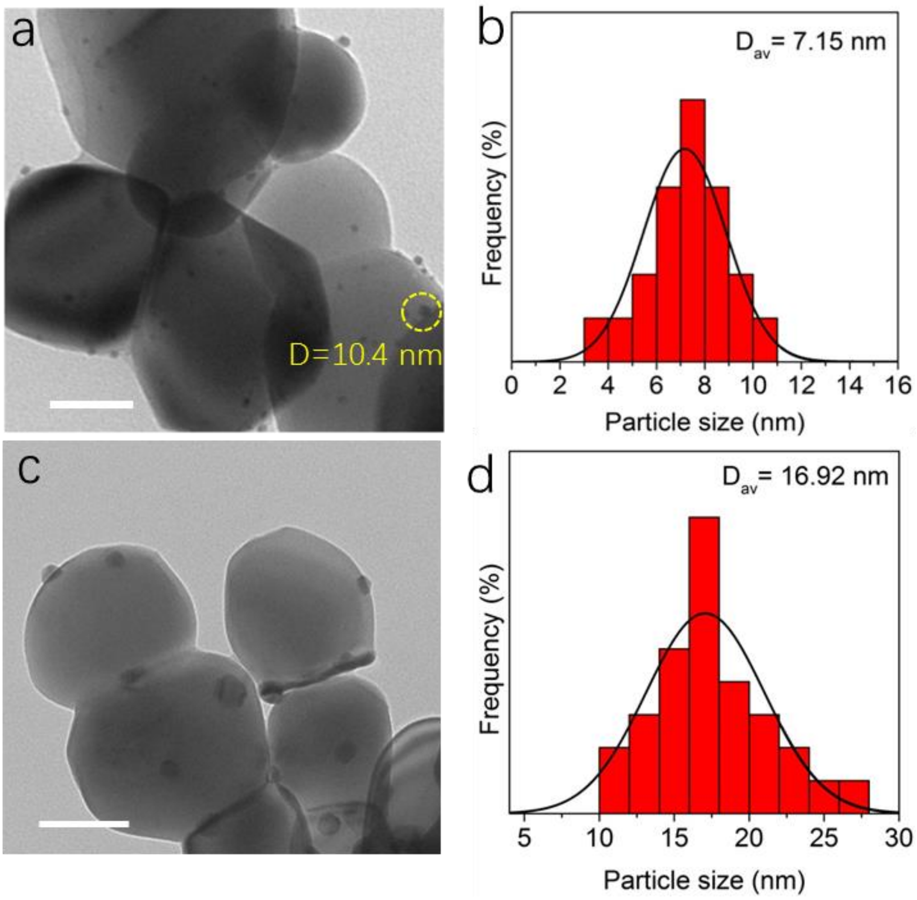

Supplementary Figure 13. Morphology characterization. (a) TEM and (b) particle-size distribution images Pd NPs/TiO_2, (c) TEM and (b) particle-size distribution images Pd NPs/TiO_2-900 after calcination at 500°C in air for 3h. Scale bar, 100 nm.

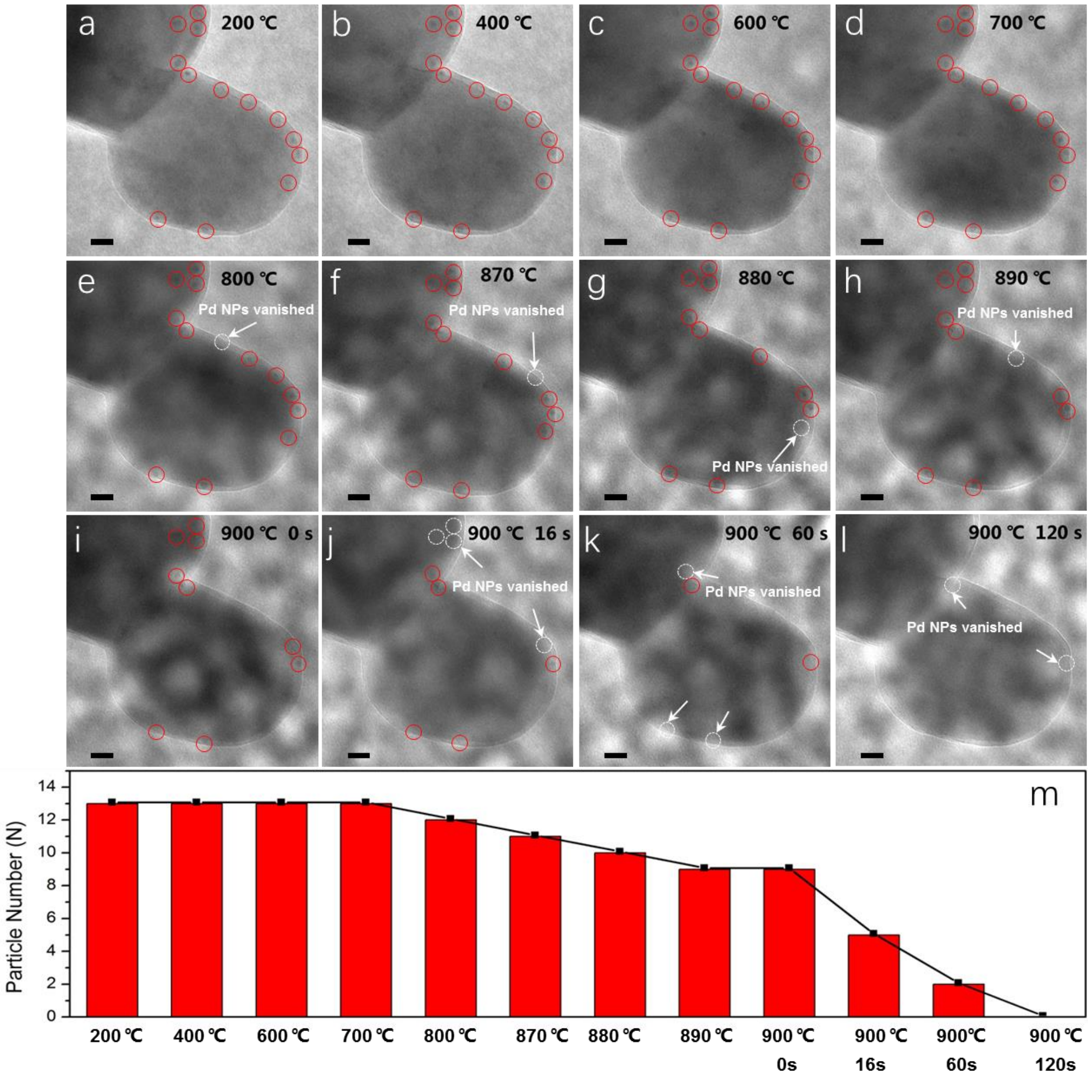

Supplementary Figure 14. Representative in-situ TEM images of Pd NPs/TiO_2@PDA-Pd NPs/TiO_2@C. (a-h) Different temperatures, (i-l) different times at 900 °C, (m) the corresponding statistical charts of Pd-NPs number. Scale bar, 20 nm.

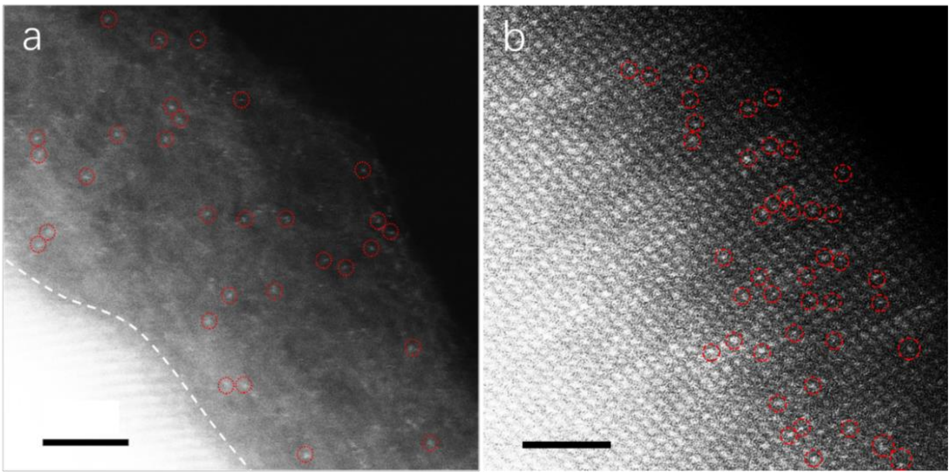

Supplementary Figure 15. Aberration-corrected HAADF-STEM images. (a) Pd SAs/TiO_2@C and (b) Pd SAs/TiO_2. The isolated bright dots (some of them are highlighted by red circles) are Pd SAs in the HAADF images. Scale bar, 2 nm.

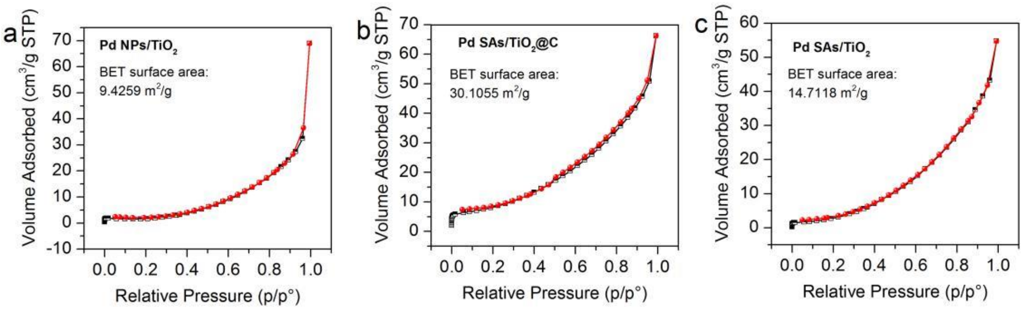

Supplementary Figure 16. N_2 adsorption–desorption isotherms and BET surface areas. (a) Pd NPs/TiO_2, (b) Pd SAs/TiO_2@C and (c) Pd SAs/TiO_2.

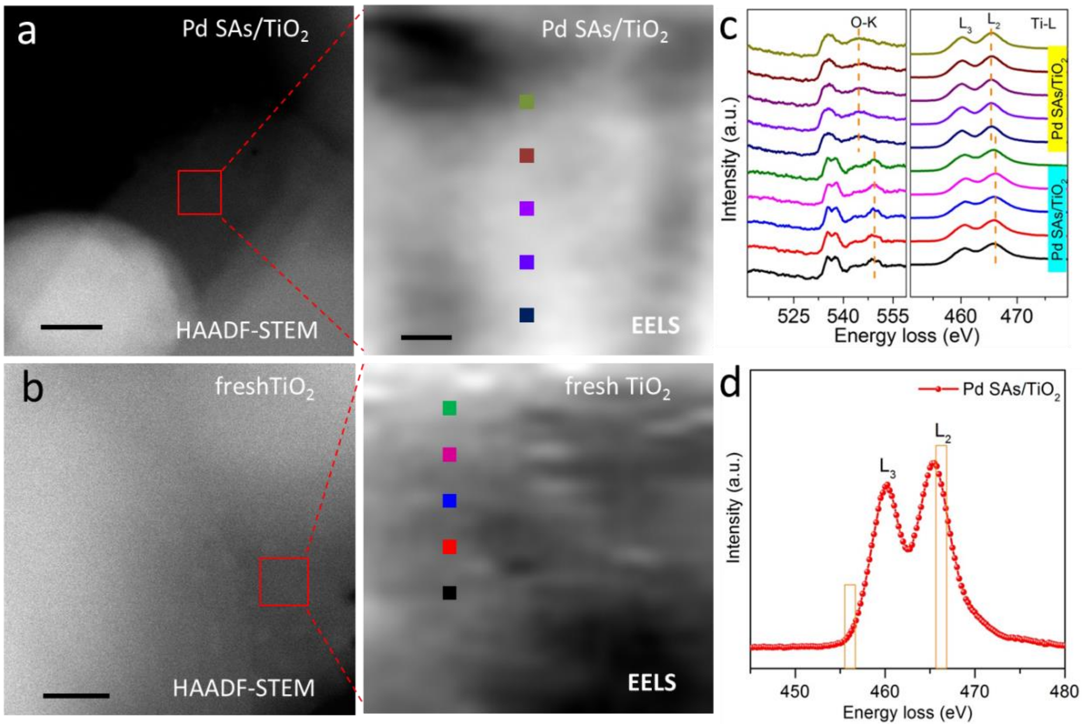

Supplementary Figure 17. Morphology and structure characterization. (a) HAADF images of Pd SAs/TiO_2. Scale bar, 10 nm (left) and 1 nm (right). (b) HAADF images of fresh TiO_2. Scale bar, 10 nm (left) and 1 nm (right). (c) Selected O−K and Ti−L EELS spectra of Pd SAs/TiO_2 and fresh TiO_2 recorded at various positions as marked by colored dots in the STEM images. (d) The ratio of the integration of both yellow areas (L_3 for Ti_{III} and L_2 for Ti_{IV}) provides information about the oxidation level of Ti cations.

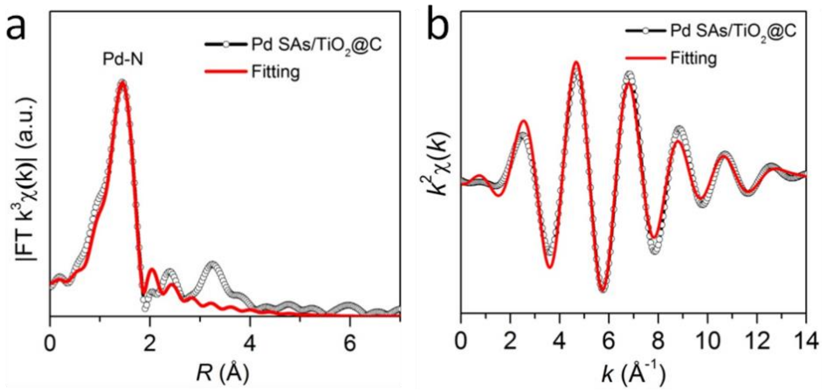

Supplementary Figure 18. EXAFS fitting curves of Pd SAs/TiO_2@C (annealing at 900°C for 3h). (a) R space and (b) k space.

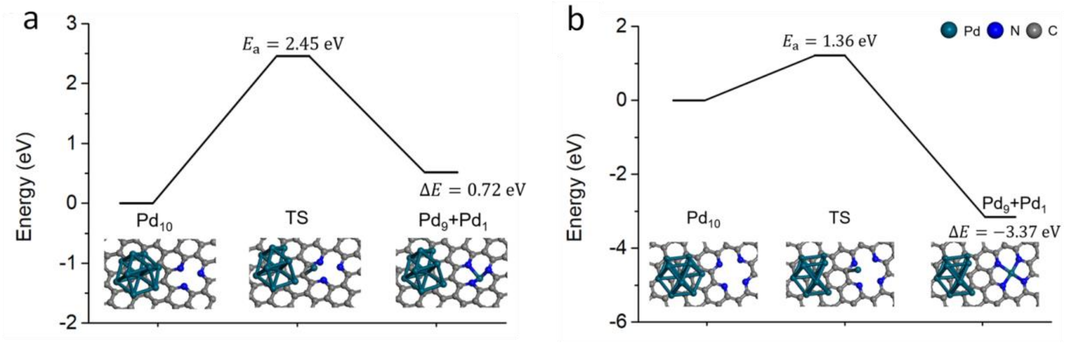

Supplementary Figure 19. Density functional theory (DFT) calculations. (a) and (b) calculated energies along the pyrolysis pathway of Pd atom for Pd_{10} cluster to Pd-N_3 and Pd-N_4 defect respectively and their corresponding configurations of initial and final states. Pd, N, and C atoms are marked in green, blue, and grey, respectively. The activation energy E_a represents the difficulty for this pyrolysis process. The total energy difference ΔE represents the stability of the final states compared with initial states which means the Pd-N_3 defect is more stable than Pd-N_4 defect.

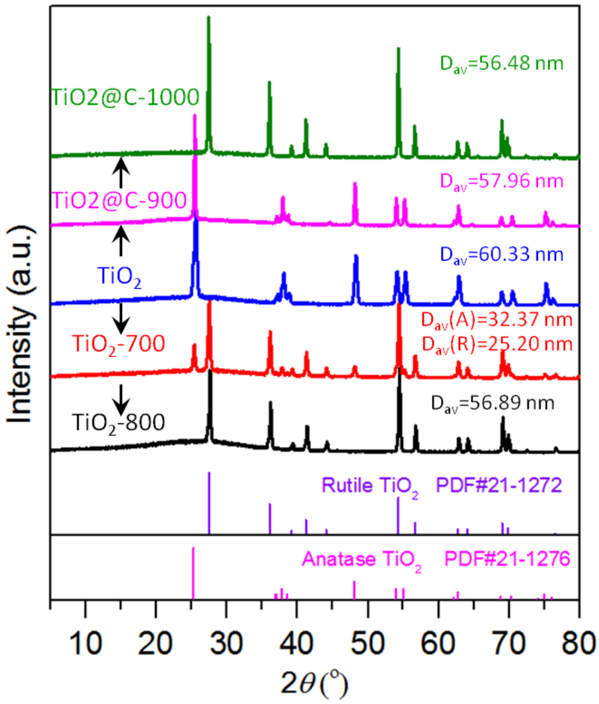

Supplementary Figure 20. XRD patterns of TiO_2 and TiO_2@PDA annealing at different temperatures. D_{av} indicates the average crystallite diameter of the anatase(A) and rutile (R) phases, which is obtained by Scherrer's law.

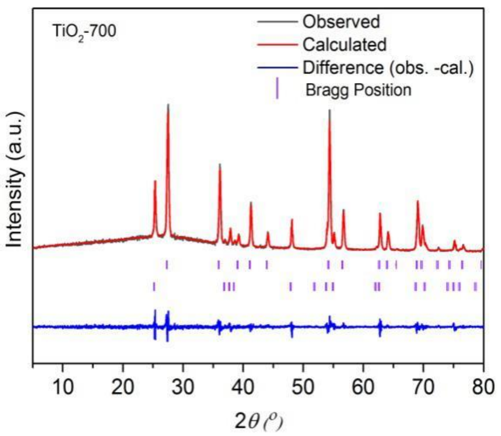

Supplementary Figure 21. Raw and calculated XRD patterns of TiO_2-700.

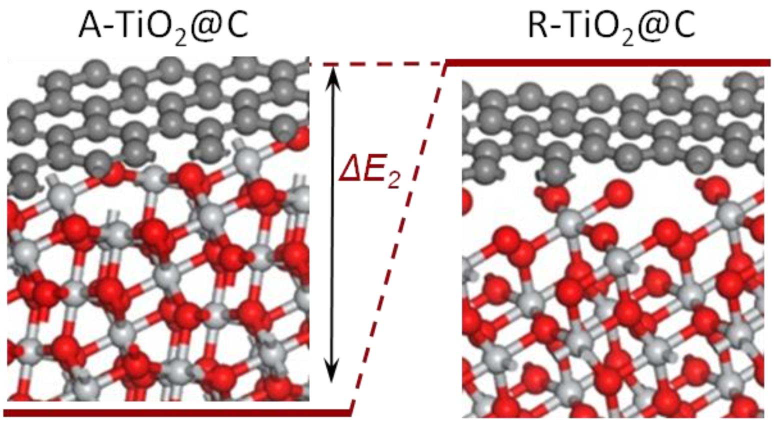

Supplementary Figure 22. Structure configurations and the energy difference of/between anatase TiO_2@carbon (A-TiO_2@C) and rutile TiO_2@carbon (R-TiO_2@C). Silver white atoms represent Ti, red atoms represent O, and grey atoms represent C.

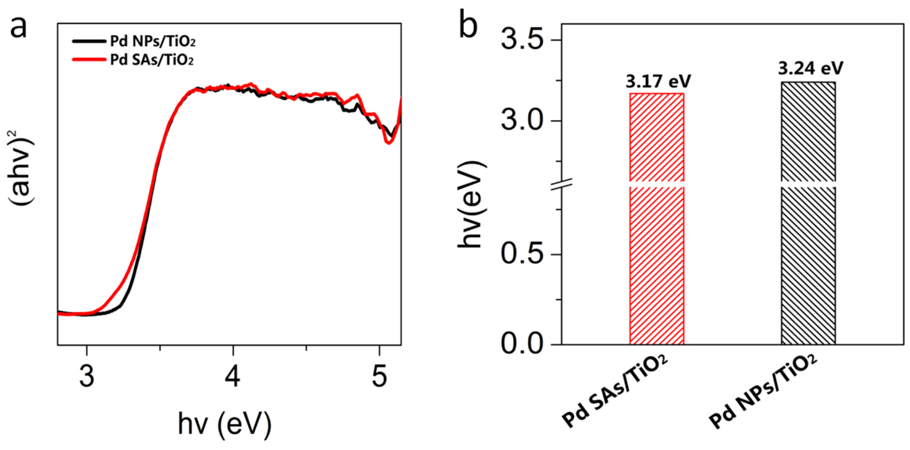

Supplementary Figure 23. UV−vis diffuse reflectance spectra. (a) Band gap images of Pd SAs/TiO_2 and Pd NPs/TiO_2, and (b) the corresponding optical band gaps.

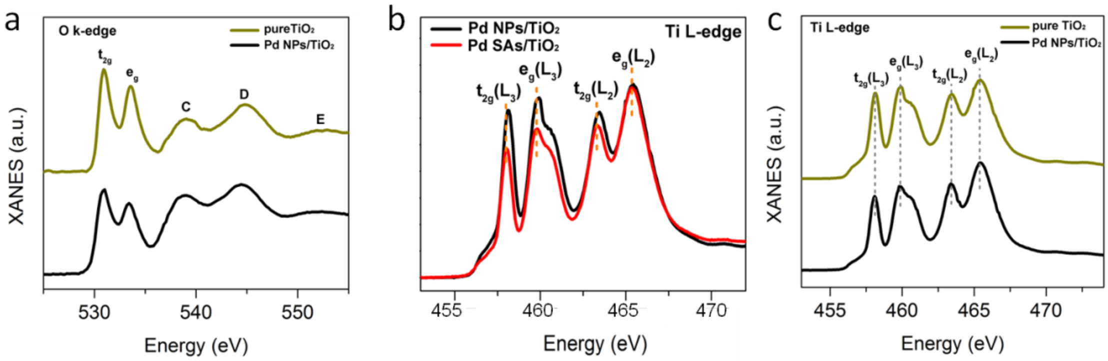

Supplementary Figure 24. XANES spectra. (a) O ls (K-edge) of pure TiO_2 and Pd NPs/TiO_2, and (b, c) Ti 2p (L-edge) of pure TiO_2, Pd NPs/TiO_2 and Pd SAs/TiO_2.

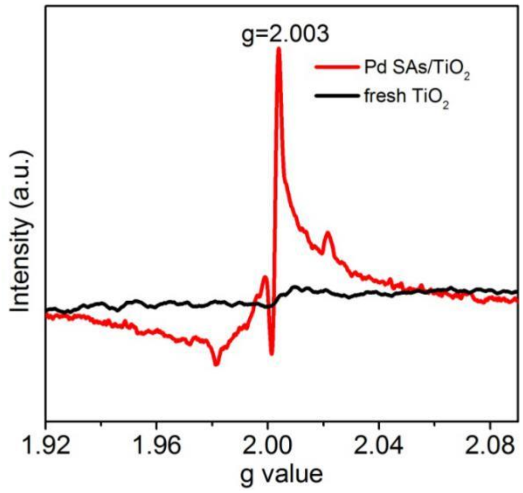

Supplementary Figure 25. EPR spectra of Pd SAs/TiO_2 and fresh TiO_2.

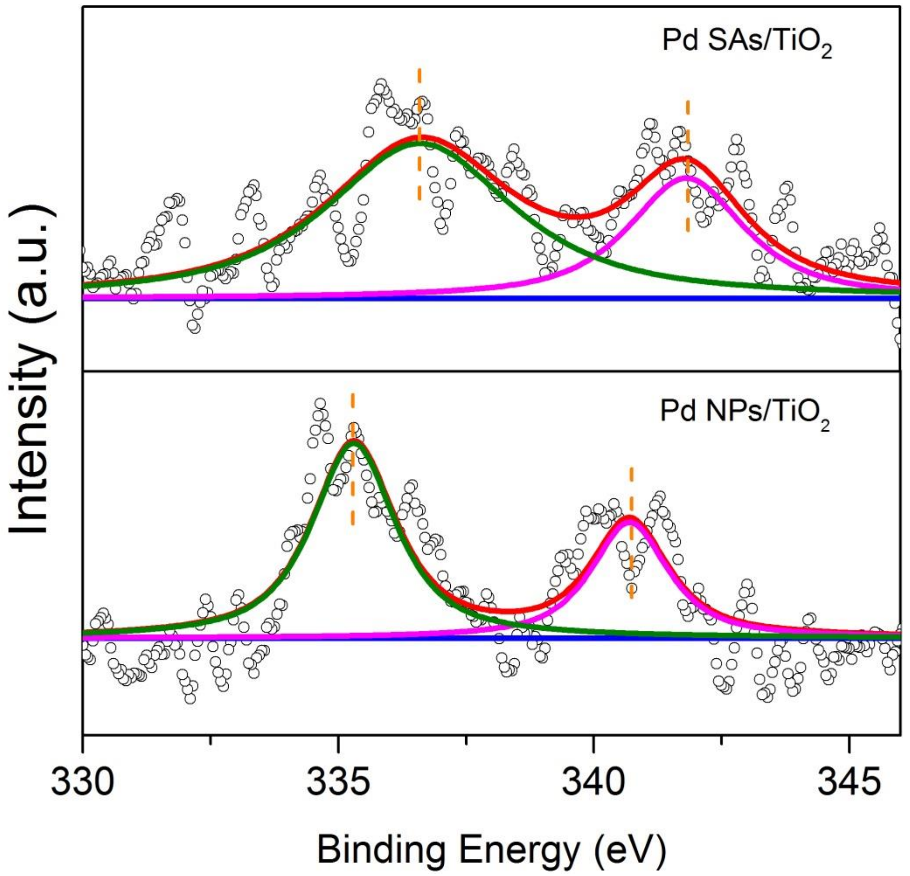

Supplementary Figure 26. XPS spectra for the Pd 3d regions of Pd SAs/TiO_2 and Pd NPs/TiO_2.

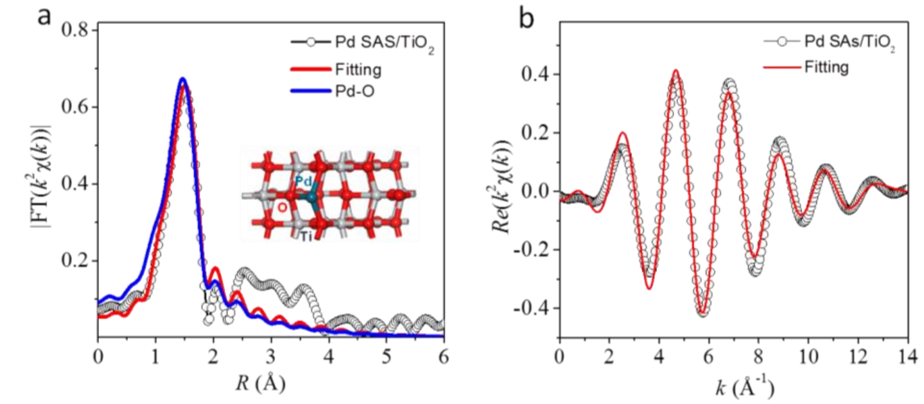

Supplementary Figure 27. EXAFS fitting curves of Pd SAs/TiO_2. (a) R space and (b) k space. Inset is the proposed Pd-O_3 architectures.

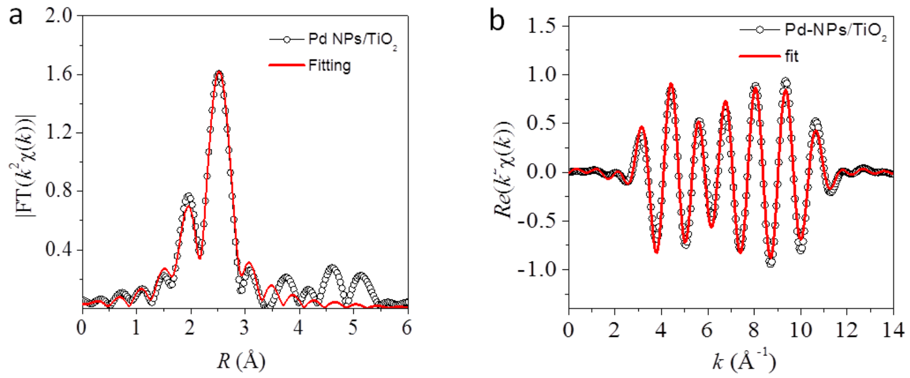

Supplementary Figure 28. EXAFS fitting curves of Pd NPs/TiO_2. (a) R space and (b) k space.

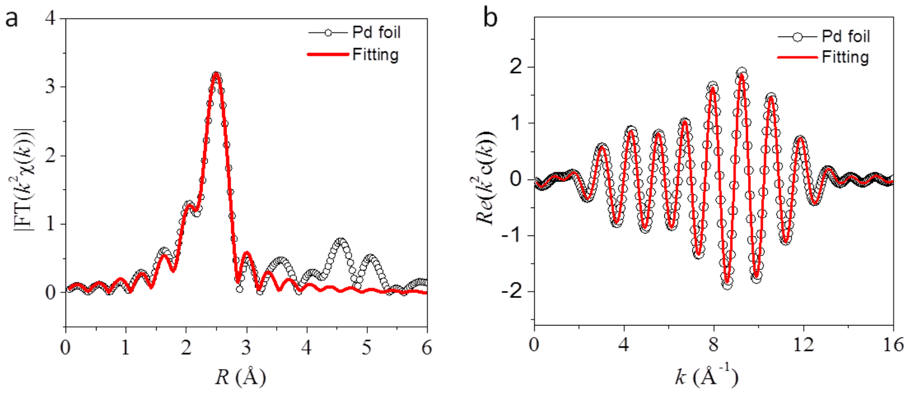

Supplementary Figure 29. EXAFS fitting curves of Pd foil. (a) R space and (b) k space.

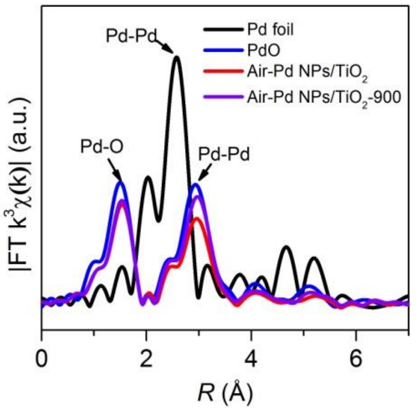

Supplementary Figure 30. Fourier transformed (FT) k^3-weighted Pd K-edge EXAFS spectra for Pd NPs/TiO_2 and Pd NPs/TiO_2-900 samples after calcination in air at 500°C.

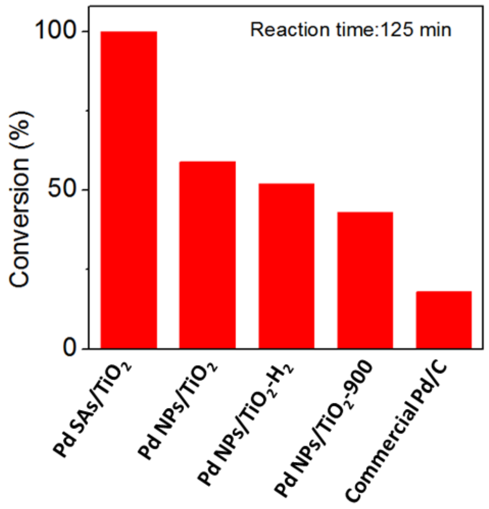

Supplementary Figure 31. Catalytic performances of the prepared Pd SAs and Pd NPs and commercial Pd/C.

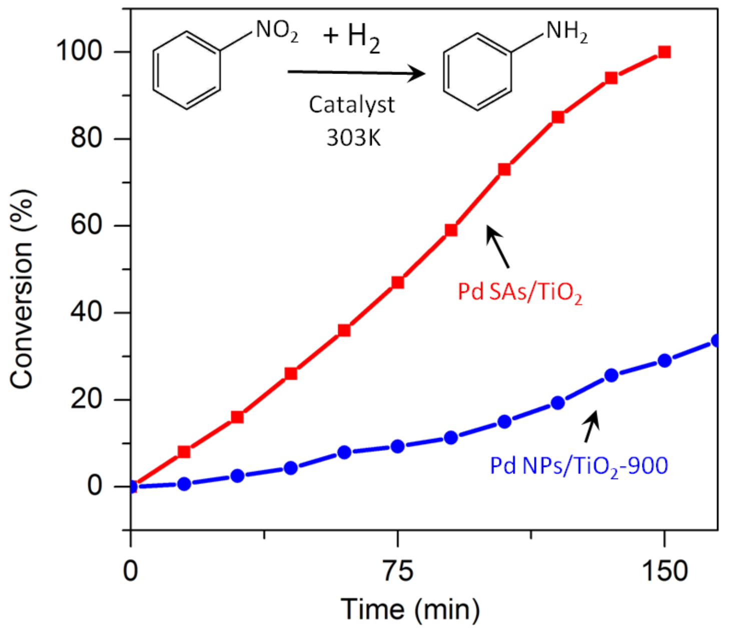

Supplementary Figure 32. Catalytic performance of Pd SAs/TiO_2 and Pd NPs/TiO_2-900 in nitrobenzene hydrogenation. Reaction condition: T = 303K; pressure = 0.1M Pa.

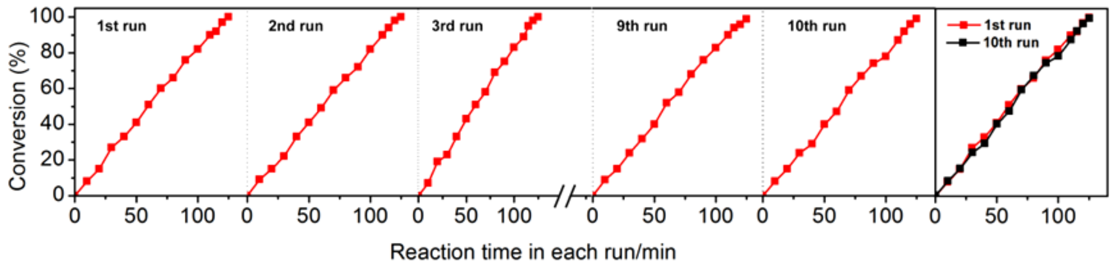

Supplementary Figure 33. Catalytic performance of Pd SAs/TiO_2 in styrene hydrogenation for 10 cycles. Reaction condition: T = 303K; pressure = 0.1M Pa.

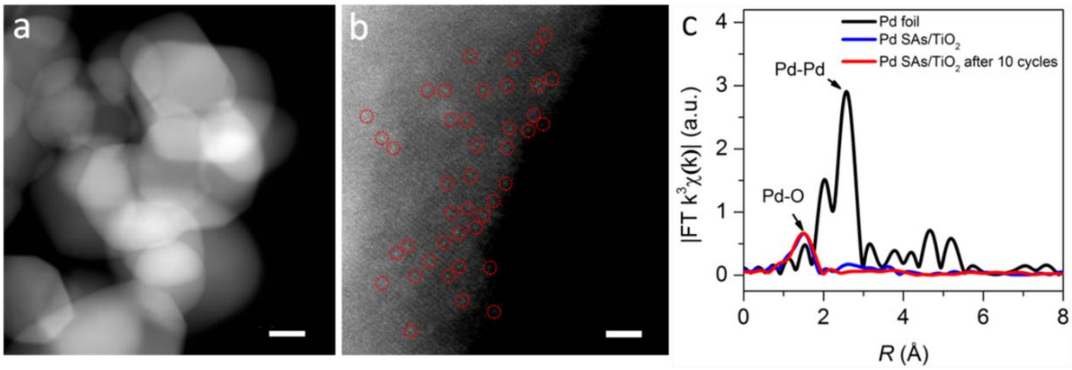

Supplementary Figure 34. Structural characterizations of Pd SAs/TiO_2 catalyst after 10 cycles reaction. (a) Low-magnification HAADF-STEM image. Scale bar, 50 nm. (b) High-resolution HAADF-STEM image. Scale bar, 1 nm. (c) Fourier transformed (FT) k3-weightedχ(k)-function of the EXAFS spectra for Pd K-edge of Pd SAs/TiO_2 before and after 10 cycles reaction. These results showed that Pd sites were still atomically dispersed after 10 cycles reaction.

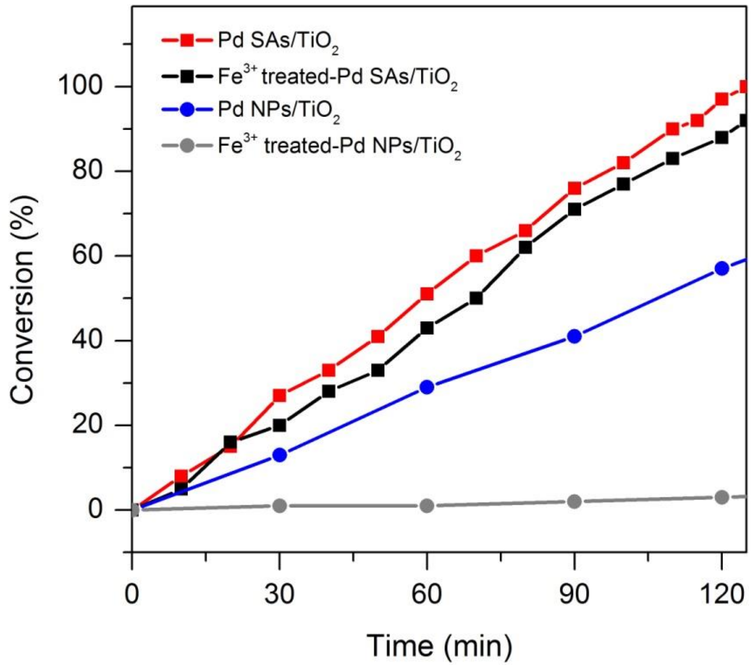

Supplementary Figure 35. Catalytic performances of Pd SAs/TiO_2 and Pd NPs/TiO_2 in styrene hydrogenation before and after treated by using an aqueous solution containing $FeCl_3$ and HCl. Reaction conditions: T = 303K; pressure = 0.1M Pa.

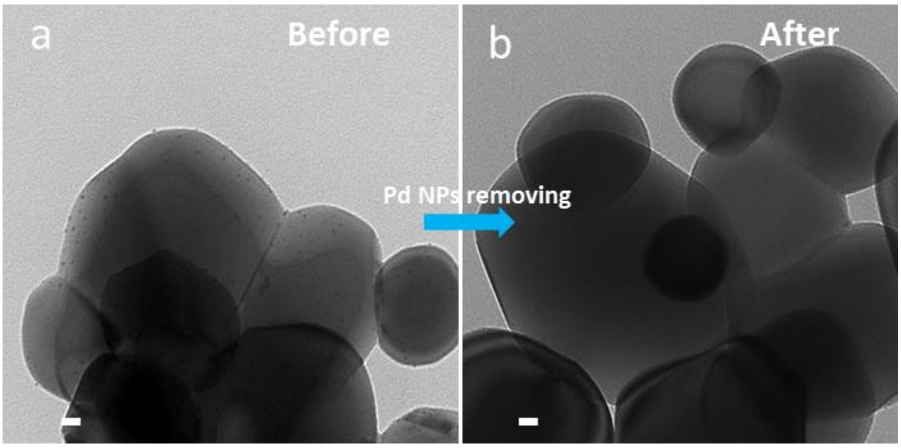

Supplementary Figure 36. Morphology characterization. TEM images of Pd NPs/TiO_2 (a) before and (b) after the etching with an aqueous solution containing $FeCl_3$ and HCl. The images in (a) and (b) showed the presence and the disappearance of the Pd NPs. Scale bar, 20 nm.

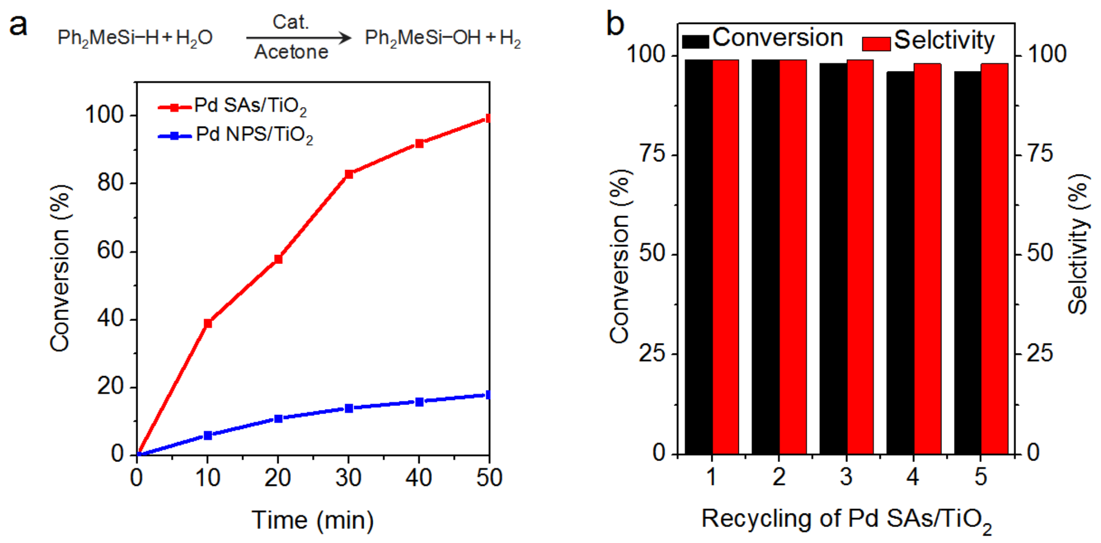

Supplementary Figure 37. Catalytic activity, stability and selectivity of Pd SAs/TiO2 in diphenylmethylsilane oxidation with water.

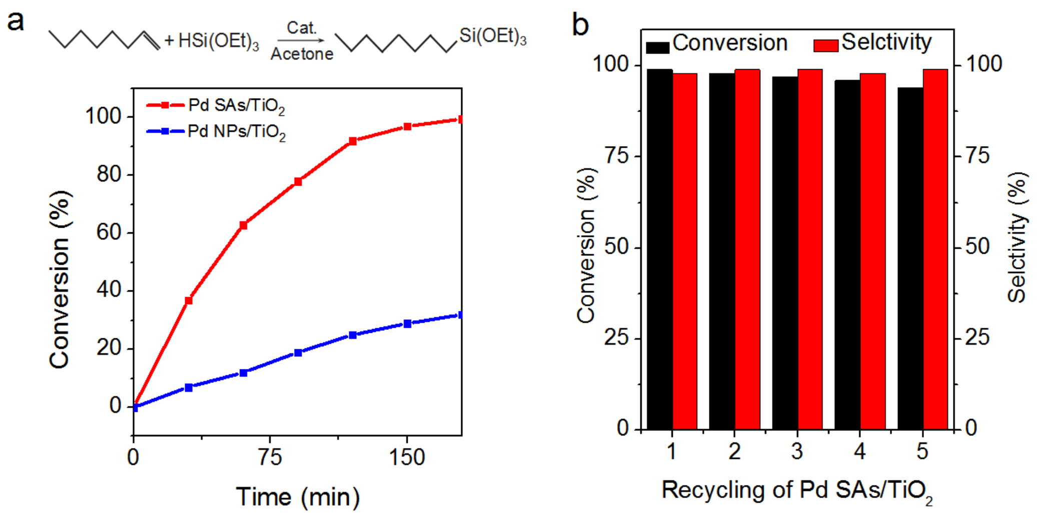

Supplementary Figure 38. Catalytic activity, stability and selectivity of Pd SAs/TiO_2 of hydrosilylation reactions with 1-octene and $(EtO)_3SiH$.

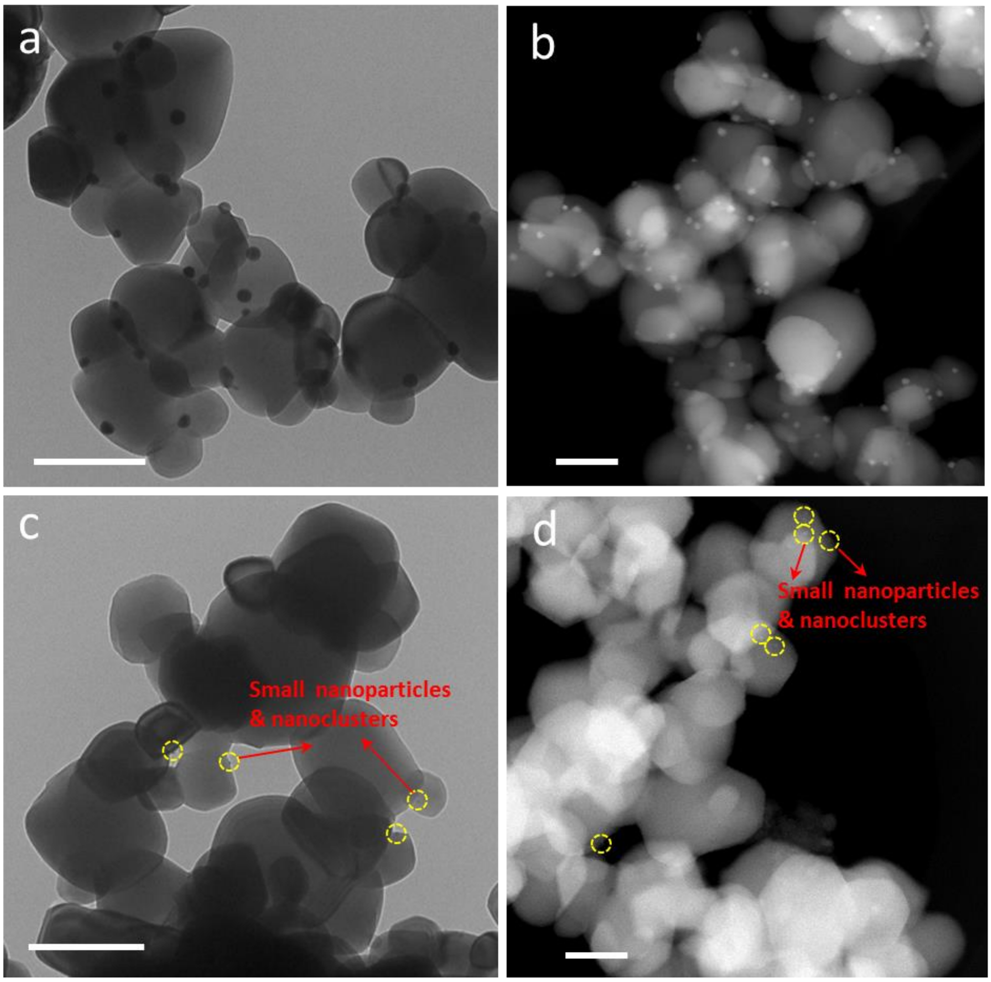

Supplementary Figure 39. Morphology characterization. Pd NPs/TiO_2-900 before (a, b) and after (c, d) treatment by thermal atomization strategy. Scale bar, 200 nm.

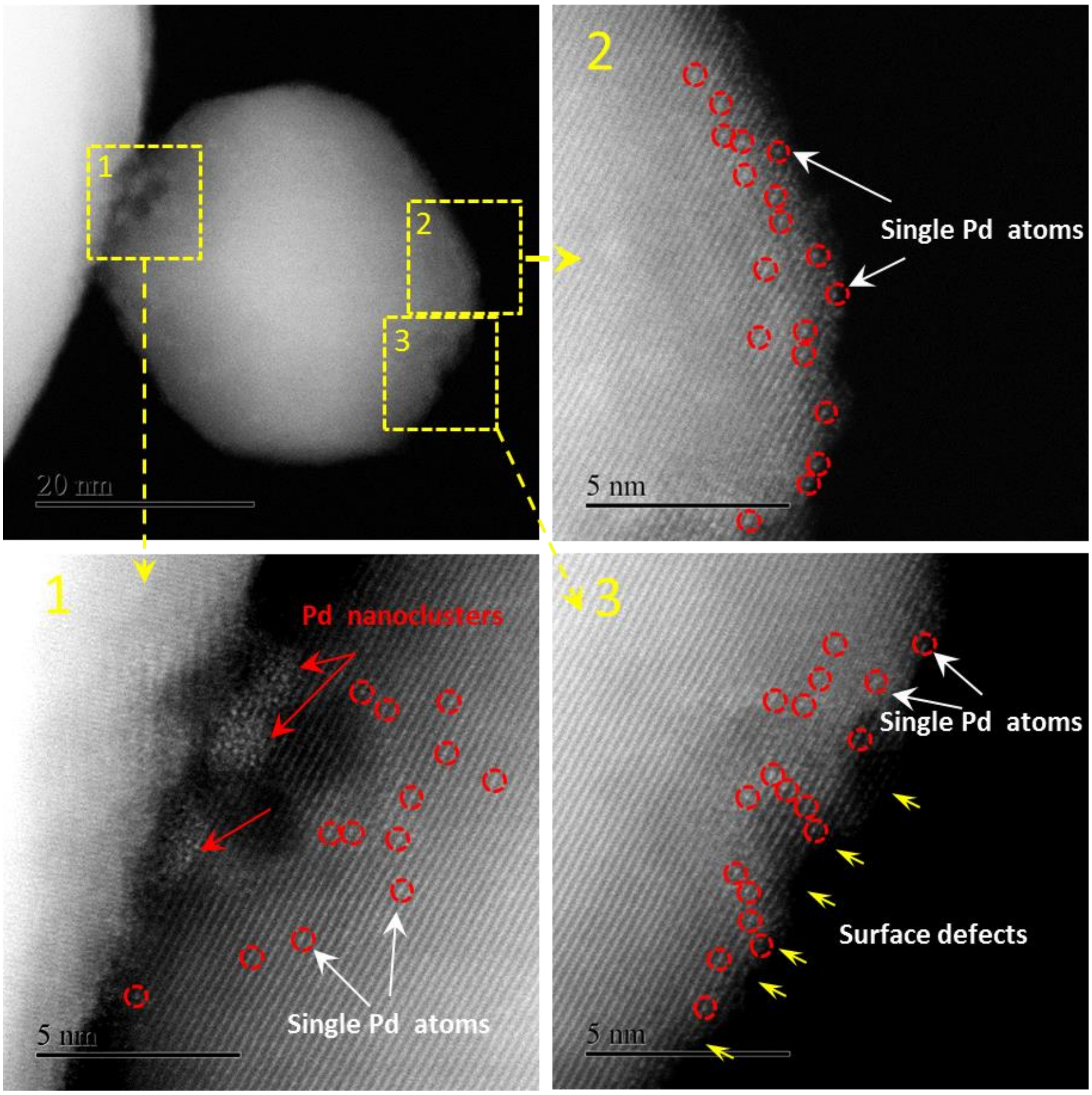

Supplementary Figure 40. High-resolution HAADF-STEM images of Pd NPs/TiO_2-900 after treatment by thermal atomization strategy. Scale bar, 20 nm for original image, 5 nm for the magnification images.

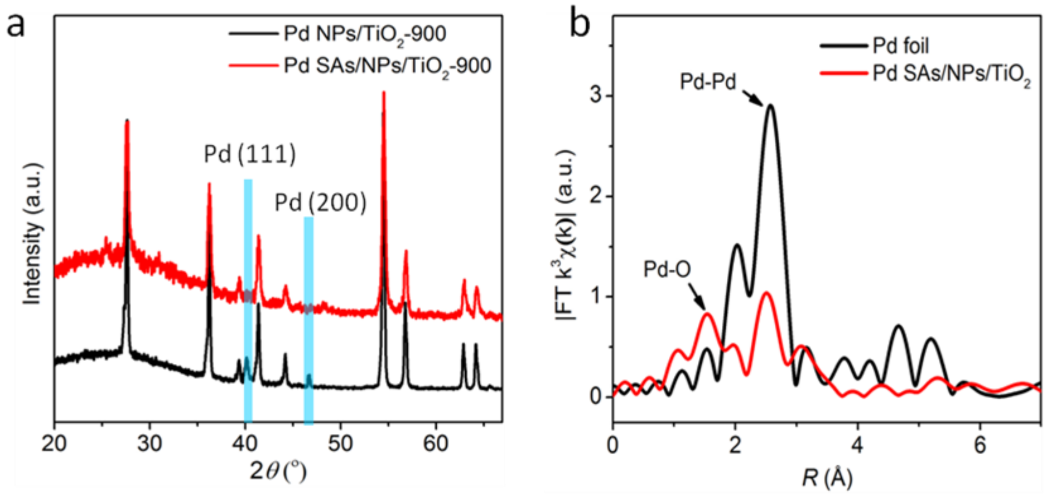

Supplementary Figure. 41 Structure characterization: (a) XRD patterns of Pd NPs/TiO_2-900 and Pd SAs/NPs/TiO_2-900. (b) Fourier transformed (FT) k3-weightedχ(k)-function of the EXAFS spectra for Pd K-edge of Pd SAs/NPs/TiO_2-900.

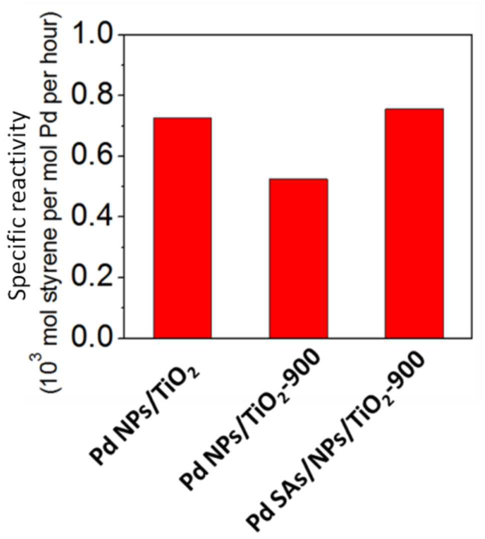

Supplementary Figure42. Specific reactivity of Pd NPs/TiO_2, Pd NPs/TiO_2-900 and Pd SAs/NPs/TiO_2-900.

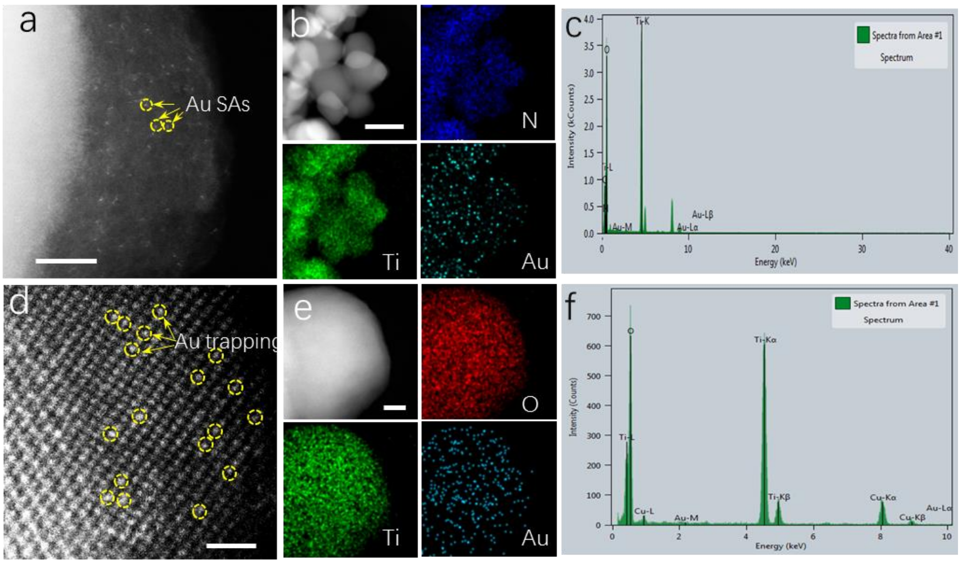

Supplementary Figure 43. Morphology characterization. (a) AC HAADF-STEM image of Au SAs/TiO_2@C. Scale bar, 5 nm. (b, c) EDS mappings and EDS spectra of Au SAs/TiO_2@C. Scale bar, 100 nm. (d) AC HAADF-STEM image of Au SAs/TiO_2. Scale bar, 1 nm. (b, c) EDS mappings and EDS spectra of Au SAs/TiO_2. Scale bar, 20 nm.

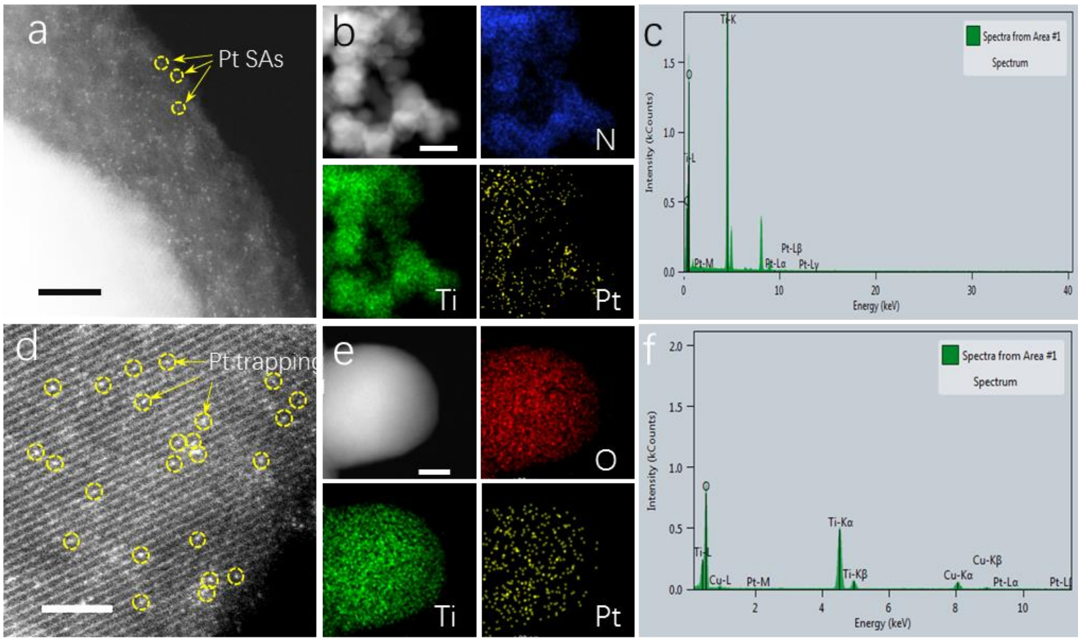

Supplementary Figure 44. Morphology characterization. (a) AC HAADF-STEM image of Pt SAs/TiO_2@C. Scale bar, 5 nm. (b, c) EDS mappings and EDS spectra of Pt SAs/TiO_2@C. Scale bar, 100 nm. (d) AC HAADF-STEM image of Pt SAs/TiO_2. Scale bar, 2 nm. (b, c) EDS mappings and EDS spectra of Pt SAs/TiO_2. Scale bar, 20 nm.

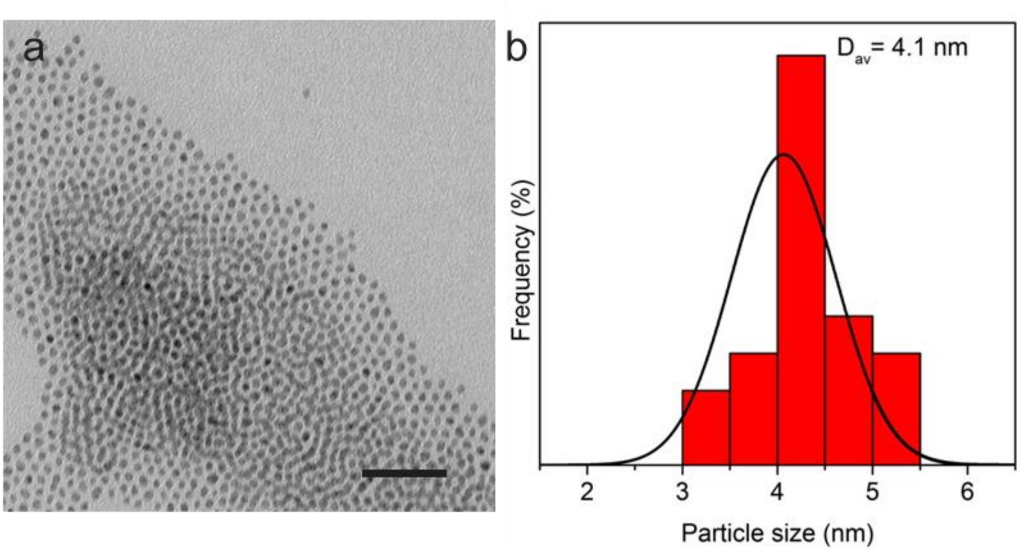

Supplementary Figure 45. Morphology characterization. (a) TEM image of Pd NPs with oleylamine coating (denoted as Pd NPs@OAm), and (b) the corresponding particle-size distribution. Scale bar, 50 nm. The prepared Pd NPs in Pd NPs@OAm were uniformly dispersed with an average diameter of 4.1 nm.

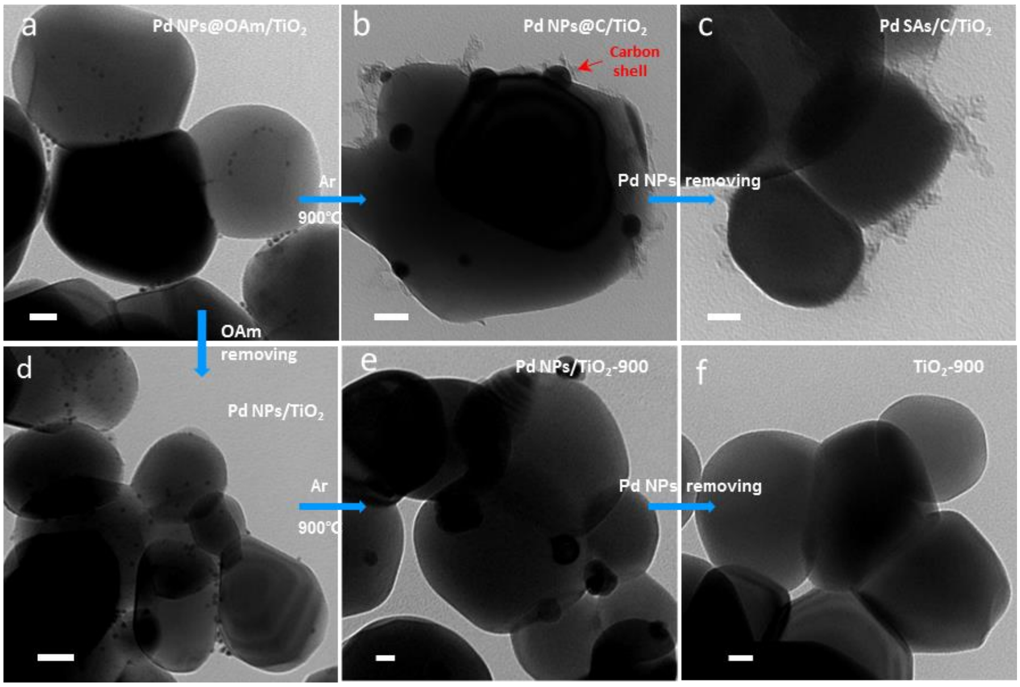

Supplementary Figure 46. TEM images characterization. (a-c) Pd species during each synthetic step by thermal atomization and the following Pd NPs removing: (a) Pd NPs@OAm/TiO_2, (b) Pd NPs@C/TiO_2, (c) Pd SAs/C/TiO_2. TEM images (d-f) of samples by conventional sintering and the following Pd NPs removing: (d) Pd NPs /TiO_2, (e) Pd NPs/TiO_2-900, (f) TiO_2-900. Scale bar, 20 nm. (b) shows the aggregation of Pd NPs and the obtained carbon shells after carbonization at 900°C. (d) shows the absence of the Pd NPs and the preservation of the carbon shells after treated by using an aqueous solution containing $FeCl_3$ and HCl. (e) shows the aggregation of Pd NPs after annealed at 900°C. (f) shows the absence of the Pd NPs after treated by using an aqueous solution containing $FeCl_3$ and HCl.

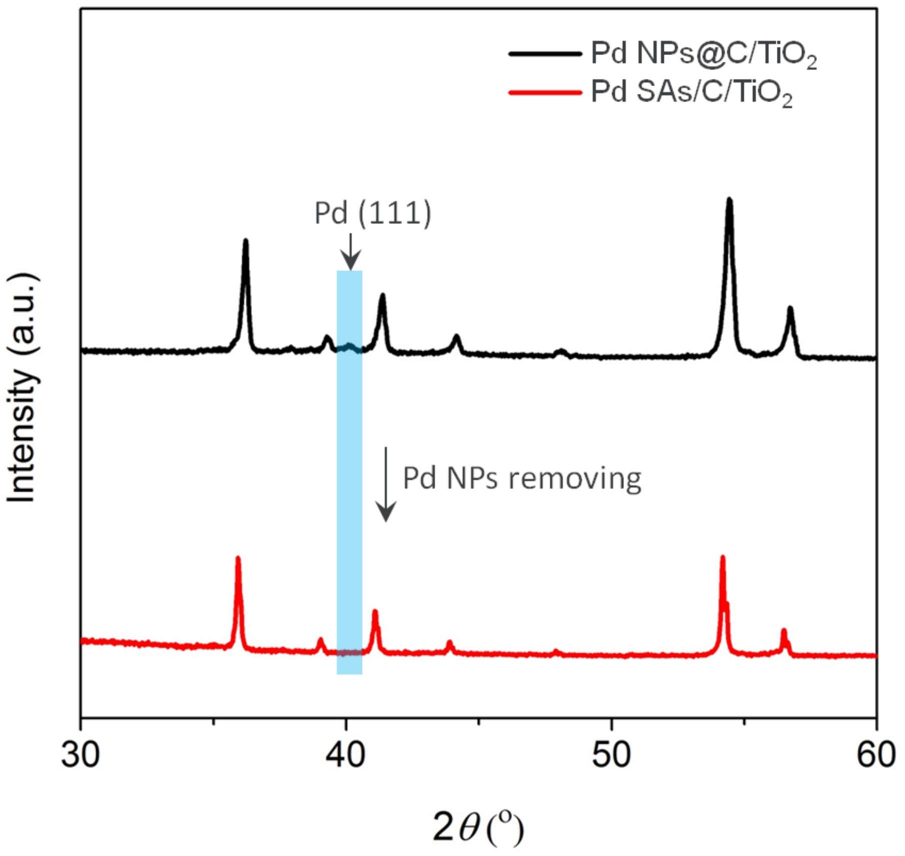

Supplementary Figure 47. XRD patterns. XRD patterns of Pd NPs@OAm/TiO_2 after carbonization at 900°C (denoted as Pd NPs@C/TiO_2) and Pd SAs/C/TiO_2, showing the vanished of Pd signals after treated by using an aqueous solution containing $FeCl_3$ and HCl.

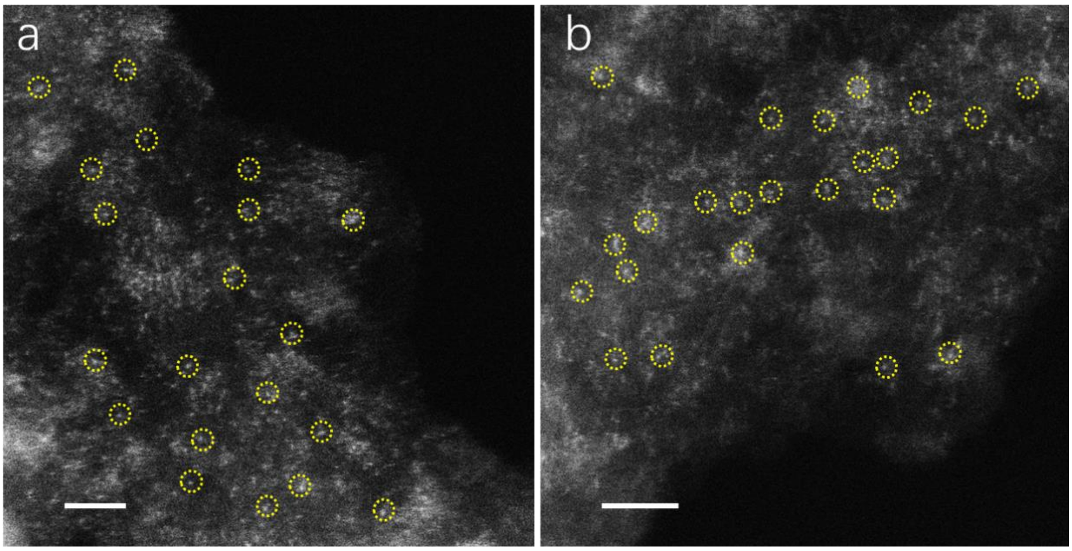

Supplementary Figure 48. AC HAADF-STEM images of Pd SAs/C@TiO_2. The OAm shells was transformed *in-situ* to nitrogen-doped carbon, in which the strong N coordination can effectively trapped Pd atoms. Scale bar, 2 nm.

Supplementary Table 1. Inductively coupled plasma optical emission spectrometry (ICP-OES) measurement

**Sample**	**The content of Pd (wt%)**
Pure TiO_2	0
Pd NPs/TiO_2	0.32
Pd NPs/TiO_2@PDA	0.31
Pd SAs/TiO_2@C	0.31
Pd SAs/TiO_2	0.30
Pd NPs/TiO_2-900	0.29
Pd SAs/NPs/TiO_2-900	0.30

Supplementary Table 2. EXAFS parameters of Pd SAs/TiO_2 and references.

**Sample**	**Path**	**N**	**R (Å)**	**σ^2 (10^{-3}Å^2)**	**ΔE_0 (eV)**	**R-factor**
Pd SAs/TiO_2	Pd-O	2.7	1.97	4.2	3.0	0.015
Pd NPs/TiO_2	Pd-Pd	9.7	2.72	8.8	-1.2	0.005
Pd SAs/TiO_2@C	Pd-N	3.6	1.97	5.8	-0.2	0.003
Pd foil	Pd-Pd	12.0*	2.74	5.4	3.9	0.001

*N is the coordination number; R is interatomic distance (the bond length between central atoms and surrounding coordination atoms); σ^2 is Debye-Waller factor (a measure of thermal and static disorder in absorber-scatterer distances); ΔE_0 is edge-energy shift (the difference between the zero inetic energy value of the sample and that of the theoretical model). *R* factor is used to value the goodness of the fitting. * This value was fixed during EXAFS fitting, based on the known structure of Pd. Error bounds that characterize the structural parameters obtained by EXAFS spectroscopy were estimated as N ± 20%; R ± 1%; σ^2 ± 20%; ΔE_0 ± 20%.

Supplementary Table 3. Refined parameters by FullProf

Sample	Anatase	Rutile	R_p	R_{wp}	χ^2
TiO_2-700	23.28 %	76.72%	1.30	1.15	1.78

Supplementary Notes

We used N-free Resorcino-formaldehyde (RF) resin as the coating polymers (Supplementary Figure 8) to replace polydopamine (with N element). After annealing at 900 °C(N_2) for 3 h, the coated RF layers were transformed *in-situ* into carbon layers (without N-doped). Meanwhile, the small Pd NPs (D_{av}=3.4 nm) on TiO_2 surface were sintered to larger Pd NPs (D_{av}=12.04 nm, Supplementary Figure 9c), as revealed by the TEM and HAADF images (Supplementary Figure 9a and b). AC HAADF-STEM (Supplementary Figure 9d-i) at different region of the carbon layers further revealed that only a few number of Pd atoms were trapped, indicating the poor anchoring ability of the N-free carbon. In contrast, when N-containing polymers (polydopamine and oleylamine) were used to coat Pd NPs/TiO_2, a large amount of Pd atoms could be observed in polymers derived N-doped carbon (Fig. 2b and –Supplementary Figure 48). These results all demonstrated that N is essential for transformation of Pd NPs to SAs.

X-ray photoelectron spectroscopy (XPS) and the near edge X-ray absorption fine structures (NEXAFS) were further performed to investigate the chemical environments of N in Pd SAs/TiO_2@C. XPS spectrum of N_{1s} in (a) revealed that two prominent peaks assigned to pyri-N (398.5eV) and graph-N (401.2 eV) were existed in Pd SAs/TiO_2@C (Supplementary Figure 10a). The pyri-N contributes one p-electron to the π conjugated system, which would facilitate the anchoring of Pd SAs[13]. More evidence can be found from the N K-edge NEXAFS spectrum in Supplementary Figure 10b, in which three peaks at 398.3, 401.1 and 407.3 eV can be assigned to the π* excitations of pyri-N and graph-N species and σ* excitation of C-N bonds[14], respectively.

We calcined the fresh Pd NPs/TiO_2 sample and Pd NPs/TiO_2-900 sample at 500°C in air for 3h to investage the change of the particle size (Supplementary Figure 13). TEM and particle-size distribution images reveal that the fresh Pd NPs of Pd NPs/TiO_2 sample with an average diameter of 3.40 nm sintered into larger particles (with an

average diameter of 7.15 nm) after annealing at 500°C in air for 3h. Similar results also can be obtained for Pd NPs/TiO_2-900 sample after thermal treatment, in which the average diameter of Pd NPs increased from 14.53 to 16.92 nm. The increase of Pd NPs size can be ascribed to Ostwald ripening or particles migration according to previous reports[15,16].

Electron energy-loss spectroscopy (EELS) provides information on both the O and Ti states from the O-K (O 1s→2p) and Ti-L (Ti 2p→3d) core edges. To further reveal the existence of abundant O vacancies, selected Ti−L and O−K electron energy-loss spectroscopy (EELS) spectra for Pd SAs/TiO_2 were recorded at various positions in the red rectangle of the STEM image (Supplementary Figure 19a). For comparison, the corresponding spectra of fresh TiO_2 were also recorded (Supplementary Figure 19b). The Ti−L edge provides a fingerprint of the Ti^{4+} and Ti^{3+} states, and the O−K edge is sensitive to the presence of oxygen vacancies. As displayed in Supplementary Figure 19c, the peak of O-K for Pd SAs/TiO_2 shifts about 1.1 eV in comparison with fresh TiO_2, indicating the generation of O defects of the former.[17] In addition, the change of Ti−L energy can also be observed (Supplementary Figure 19c), demonstrating the existence of Ti^{3+} in the Pd SAs/TiO_2. The rate of Ti^{3+} was further calculated to ca. 16.6% (Supplementary Figure 19d) based on the reported method.[18] The surface oxygen vacancies often have two electrons, and some of them are reduced by the adjoining Ti^{4+} to Ti^{3+}. So, the formation of Ti^{3+} also verified the existence of surface O vacancies indirectly[19].

Quantitative analysis of the sample after annealing at 900°C for 3h (Pd SAs/TiO_2@C) was carried out through least square EXAFS fitting. The fitting curves are displayed in Supplementary Figure 18, and the corresponding structure parameters are listed in Table S2. According to the fitting results, the proposed local structure of Pd SAs involves coordination by four N.

Furthermore, density functional theory (DFT) calculations were performed to simulate the process of thermal atomization (Supplementary Figure 19). Both N_3 (a) and N_4 (b) defects were considered. We adopted Pd_{10} as an example and found that spliting a single Pd atom from the Pd_{10} cluster by the coordination of N_3 requires to overcome a big barrier (2.45 eV), and an endothermicity reaction of +0.72 eV. However, a Pd-N_4 moieties only requires 1.36 eV to overcome, followed by a large exothermicity of 3.37 eV. These results indicate that the single Pd atom trapped by N_4 defect is more stable than the Pd atom trapped by N_3 defect. Thus, a single Pd atom in Pd SAs/TiO_2@C is more likely to coordinate with four nitrogen atoms, agreeing well with the local structure obtained by XAFS.

We observed that the anatase TiO_2 used in our experiment would completely transform into rutile TiO_2 when the calcination temperature over 700°C. As shown in Supplementary Figure 20, a mixed phase (anatase and rutile) appeared when the temperature up to 700°C. The relative amounts for anatase and rutile are 23.28% and 76.72%, respectively (obtained by FullProf, seen in Supplementary Figure 21 and Table S3). Anatase TiO_2 used in our experiment would completely transform into rutile TiO_2. As the temperature was elevated up to 800°C, only rutile phase was observed from XRD pattern, indicating the phase transformation had been finished. However, when the anatase TiO_2 was coated by carbon layers, the phase transformation indeed was not observed even the temperature up to 900°C. But a higher temperature (1000°C) could lead the anticipative phase transformation from anatase to rutile. These results reveal that the carbon layers on TiO_2 can prevent or delay phase transformation. Furthermore, the sizes of the anatase and rutile crystallites at different temperature were calculated according to Scherrer's law (Supplementary Figure 21).

When the carbon layer (without carbon) was coated on anatase TiO_2 (Supplementary Figure 22), the process of phase transition exhibits an endothermicity reaction with a ΔE of +4.88 eV, also indicating that the phase transition of TiO_2@C needs more external energy in comparison with pure TiO_2(ΔE=-1.44 eV).

The Pd NPs/TiO_2 sample and Pd NPs/TiO_2-900 samples after calcination in air at 500^oC were characterized by EXAFS (Supplementary Figure 30). Both EXAFS curves of Pd NPs/TiO_2 and Pd NPs/TiO_2-900 show two prominent characteristic peak at around 1.5 Å and 3.0 Å, corresponding to Pd-O and Pd-Pd bonds, respectively. Also, these curves are similar to the reference of PdO, indicating the Pd NPs on TiO_2 were transformed to PdO after calcination in air at 500^oC for 3h.

With the same reaction time (125 min) and comparable metal loading (Supplementary Figure 31), the Pd SAs/TiO_2, Pd NPs/TiO_2-H_2 and Pd/C delivered >99%, 51% and 18% conversion, indicating the highest activity of Pd SAs.

TEM and STEM images of Pd NPs/TiO_2-900 in Supplementary Figures 39 and 2 showed that many Pd NPs sintered severely with average diameter up to ~14.53 nm. Interestingly, after treatment by thermal atomization process, all large Pd NPs vanished as shown in TEM and STEM images (c, d), only remaining few nanoclusters (~ 2 nm). These results demonstrate that most of large Pd NPs may transform to atomic Pd species.

High-resolution HAADF-STEM images (Supplementary Figure 40) of Pd NPs/TiO_2-900 after treatment by thermal atomization process (denoted as Pd SAs/NPs/TiO_2-900) showed that few Pd nanoclusters are existed at surface of TiO_2 (where red arrows are pointing in region 1), while a large amount of Pd single atoms are dispersed over the whole architecture (where red circles indicate Pd single atoms). These results indicate that large Pd NPs of Pd NPs/TiO_2-900 are mainly transformed into atomic Pd species after treatment by thermal atomization process. Also, high-resolution HAADF-STEM image showed that many defects are existed at surface of TiO_2 (where white arrows are pointing in region 3), which are conductive to the stabilization of atomic Pd species.

X-ray diffraction (XRD) patterns (Supplementary Figure 41) displayed that the characteristic peaks intensity of Pd (111) at ~40.2°, Pd (200) at ~46.6° decreased or

disappeared after the thermal atomization (from Pd NPs/TiO_2-900 to Pd SAs/NPs/TiO_2-900), indicating the evolution from large Pd NPs to small NPs or atomic species. EXAFS spectra of Pd SAs/NPs/TiO_2-900 displayed a peak around 2.5 Å and a peak around 1.5 Å, which were attributed to Pd-Pd bond and Pd-O bond, respectively. This verified the co-existense of Pd NPs and SAs in Pd SAs/NPs/TiO_2-900, indicating the successful transformation of Pd NPs to SAs during the thermal atomization process.

To test the generality of our strategy, small Au/Pt NPs were supported on TiO_2 surface and coated by PDA layers. Such Au/Pt NPs could be successful transformed into atomically dispersed Au/Pt after annealing (1000°C) due to the strong coordination of N, as demonstrated by the AC HAADF-STEM and EDS mapping images in the carbon layers (Supplementary Figures 43a and b; Supplementary Figures 44a and b). Also, Au/Pt atoms were also trapped by O defects on TiO_2 surface after removing the carbon layers (Supplementary Figures 43d and 44d). EDS mapping images reveal that the Au/Pt, O and Ti are homogeneously dispersed over the whole architecture (Supplementary Figures 43e and 44e). These results all reveal that the supported Au/Pt NPs can be re-dispersed to atomic metal species by our strategy.

Supplementary methods

Computational methods. The Vienna Ab-initio Simulation Package (VASP) code was used to perform density functional theory (DFT) calculations[1-3]. The projector augmented wave (PAW) method was used to describe the interaction between the valence electrons and ionic cores[4,5]. The optB88-vdW method was utilized as the exchange-correlation functional that accounts for the long-range van der Waals (vdW) forces[6-9]. This functional is a revised version of the vdW-DFT functional, which has been demonstrated to improve the accuracy for a variety of molecules on transition-metal surfaces.

The equilibrium lattice constants of anatase which has a tetragonal structure are $a = b = 3.79$ Å, $c = 9.53$ Å and the lattice constants are in good agreement with previous experiments[10]. Using the corrected lattice constants, we established two slab models based on (101) surface which attracts attentions recently for anatase. To eliminate the polarity of TiO_2, we set O as the top and bottom layer whose position are almost the same. One is the pure (101) surface slab supercell structure which contains 88 atoms with 22 layers. The other is based on the pure (101) surface slab supercell structure and it is covered with one nitrogen-doped carbon (NC) layer (see Fig. X). This structure includes 88 atoms for anatase, 29 atoms for C and one atom for N with 2.2% strain on NC layer. In order to ensure the accuracy of our coverage structure, we have also tested the other three positions of N atom. The results have shown that the different N atom positions have little effect on the structure, this may account for the chemical environment of the top (101) layer is similar. A 15 Å vacuum was set to eliminate the interaction between slabs. The Brillouin zone was sampled with a $5 \times 3 \times 1$ k-point mesh along with the energy cutoff of 500 eV[11]. For pure (101) surface structure, the uppermost 10 layers were allowed to relax while the bottom 12 layers were fixed. As to the structure covered with NC layer, the relaxation setting is the same as that of pure (101) surface structure besides the top NC layer were also relaxed. A residual force threshold of 0.001 eV/Å was used for geometry optimizations. Convergence tests shown that increasing the accuracy settings has little effect on the calculation results.

As to the TiO_2 rutile phase, which has a tetragonal structure with lattice constants of

$a = b = 4.53$ Å, $c = 2.92$ Å. The pure (101) surface slab supercell structure contains the same number of atoms as that of anatase. For the structure covered with NC layer, it only includes 17 C atoms and 1 N atom with 7.2% strain on NC layer. The Brillouin zone was sampled with a $5 \times 9 \times 1$ k-point mesh. Other calculation settings are the same as that of anatase system.

Phase transition processes are always corresponding to energy change. In order to calculate the energy difference between the two phases, we set two comparison calculations: (1) pure (101) surface of anatase and rutile. (2) (101) surface of anatase and rutile covered with NC layer. We define the energy difference between anatase(A) and rutile (R) as $\Delta E_1 = E_R - E_A$, where the E_R and E_A represent the total energy of anatase and rutile supercell respectively. For the structures covered with NC layers, due to their atom numbers are different, then the equation can be modified as:

$$\Delta E_2 = E_{R@NC} - E_{A@NC} + E_G \qquad (1)$$

where the $E_{R@NC}$ and $E_{A@NC}$ represent the total energy of supercells of anatase and rutile covered with a NC layer. The E_G which represents the total energy of a graphene containing 12 atoms was included for a more reliable comparison between the different chemical forms[12]. For the structures covered with pure carbon layers, the equation can be modified as:

$$\Delta E_3 = E_{R@C} - E_{A@C} + E_G \qquad (2)$$

where the $E_{R@C}$ and $E_{A@C}$ represent the total energy of supercells of anatase and rutile covered with a carbon layer. The E_G which represents the total energy of a graphene containing 12 atoms was included for a more reliable comparison between the different chemical forms.

We use the optB88-vdW method to do the TS search. Brillouin zone was sampled with a 3×3×1 k-point mesh. We established slab models based on the 8×8 graphite, and a 15 Å vacuum was set to eliminate the interaction between slabs. In order to ensure the stable adsorption position of Pd_{10} cluster, we tested the different positions around the defect. The results show that the displayed structure possesses a stable state. The structures of Pd-N_3 defect and Pd-N_4 defect contain 137 atoms (124 C atoms) and 136 atoms (122 C atoms) respectively. The energy cutoff of 500 eV and a residual force

threshold of 0.001 eV/Å was used for geometry optimizations.

Supplementary References

1. Kresse, G. & Hafner, J. Ab initio molecular dynamics for liquid metals. *Phys. Rev. B Condens. Mat.* **48**, 13115-13118 (1993).
2. Kresse, G. & Hafner, J. Norm-conserving and ultrasoft pseudopotentials for first-row and transition elements. *J. Phys-Condens. Mat.* **6**, 8245-8257 (1999).
3. Kresse G, & Furthmüller, J. Efficient iterative schemes for ab initio total-energy calculations using a plane-wave basis set. *Phys. Rev. B* **54**, 11169-11186 (1996).
4. Perdew, J. et al. Generalized gradient approximation made simple. *Phys. Rev. Lett.* **77**, 3865-3868 (1998).
5. Kresse, G & Joubert, D. From ultrasoft pseudopotentials to the projector augmented-wave method. *Phys. Rev. B* **59**, 1758-1775 (1999).
6. Klimes, J., Bowler, D. & Michaelides, A. Chemical accuracy for the van der Waals density functional. *J. Phys-Condens. Mat.* **22**, 022201 (2010).
7. Klimes, J., Bowler, D. & Michaelides, A. Van der Waals density functionals applied to solids. *Physical Review B* **83,** 195131 (2011).
8. Alexandre, T., Distasio, R. A., Roberto, C. & Matthias, S. Accurate and efficient method for many-body van der Waals interactions. *Phys. Rev. Lett.* **108**, 236402 (2012).
9. Dion, M., Rydberg, H., Langreth, D. C. & Lundqvist, B. I. Van der Waals density functional for general geometries. *Phy. Rev. Lett.* **92**, 246401 (2004).
10. Graziano, G. et al. Improved description of soft layered materials with van der Waals density functional theory. *J. Phys-Condens. Mat.* **24,** 424216 (2012).
11. Monkhorst, H. J. Special points for Brillouin-zone integrations. *J. Phys-Condens. Mat.* **16**, 1748-1749 (1976).
12. Wang, Y., Yang, S., Fuentes-Cabrera, M., Li, S. & Liu, W. Enhancing Enantiomeric Separation with Strain: The Case of Serine on Cu (531). *J. Am. Chem. Soc.* **139**, 8167-8173 (2017).
13. Xin, W., Chen, W., Zhang, L., Yao, T. & Liu, W. Uncoordinated amine groups of MOFs to anchor single Ru sites as chemoselective catalysts towards the hydrogenation of quinoline. *J. Am. Chem. Soc.* **139**, 9419-9422 (2017).
14. Zhao, Y. et al. Few-layer graphdiyne doped with sp-hybridized nitrogen atoms at acetylenic sites for oxygen reduction electrocatalysis. *Nat. Chem.* **10**, 924–931 (2018).
15. Hansen, T. W., DeLaRiva, A. T., Challa, S. R. & Datye, A. K. Sintering of catalytic nanoparticles: particle migration or Ostwald ripening? *Acc. Chem. Res.* **46**, 1720-1730 (2013).

16. Simonsen, S. R. B. et al. Direct Observations of Oxygen-induced Platinum Nanoparticle Ripening Studied by In Situ TEM. *J. Am. Chem. Soc.* **132**, 7968-7975 (2010).

17. Lü, X. et al. Conducting Interface in Oxide Homojunction: Understanding of Superior Properties in Black TiO_2. *Nano Letters* **16**, 5751-5755 (2016).

18. Koketsu, T. et al. Reversible magnesium and aluminium ions insertion in cation-deficient anatase TiO_2. *Nat. Mater.* **16**, 1142–1148 (2017).

19. Li, J. et al. Synergistic effect of surface and bulk single-electron-trapped oxygen vacancy of TiO_2 in the photocatalytic reduction of CO_2. *Appl. Catal. B- Environ.* **206**, 300-307 (2017).
