# Supplementary material for: Recover the activity of sintered supported catalysts by nitrogen-doped carbon atomization
Source: Nat Commun. 2020 Jan 17;11:335. doi: 10.1038/s41467-019-14223-w (PMC6969067; doi:10.1038/s41467-019-14223-w)
Supplement: Supplementary file 2 — Description of Additional Supplementary Files [file 41467_2019_14223_MOESM2_ESM.pdf]

Description of Additional Supplementary Files

File Name: Supplementary Movie 1

Description: A movie of in-situ TEM images showed the transformation of Pd NPs to Pd SAs occurring in the temperature window from 100°C to 900°C under Ar atmosphere. The movie revealed that the migration and aggregation of Pd NPs were largely prohibited by the carbon layers, and thermal atomization would be initiated and accelerated with the temperature rise, resulting in a gradually decreasing particle number of supported Pd NPs.
